# Ciliates learn to diagnose and correct classical error syndromes in mating strategies

**DOI:** 10.3389/fmicb.2013.00229

**Published:** 2013-08-19

**Authors:** Kevin B. Clark

**Affiliations:** Research and Development Service, Veterans Affairs Greater Los Angeles Healthcare SystemLos Angeles, CA, USA

**Keywords:** courtship and dominance displays, evolutionary psychology, intracellular calcium, mate selection, mating pathways, pheromones, social decision making, soft-matter physics

## Abstract

Preconjugal ciliates learn classical repetition error-correction codes to safeguard mating messages and replies from corruption by “rivals” and local ambient noise. Because individual cells behave as memory channels with Szilárd engine attributes, these coding schemes also might be used to limit, diagnose, and correct mating-signal errors due to noisy intracellular information processing. The present study, therefore, assessed whether heterotrich ciliates effect fault-tolerant signal planning and execution by modifying engine performance, and consequently entropy content of codes, during mock cell–cell communication. Socially meaningful serial vibrations emitted from an ambiguous artificial source initiated ciliate behavioral signaling performances known to advertise mating fitness with varying courtship strategies. Microbes, employing calcium-dependent Hebbian-like decision making, learned to diagnose then correct error syndromes by recursively matching Boltzmann entropies between signal planning and execution stages via “power” or “refrigeration” cycles. All eight serial contraction and reversal strategies incurred errors in entropy magnitude by the execution stage of processing. Absolute errors, however, subtended expected threshold values for single bit-flip errors in three-bit replies, indicating coding schemes protected information content throughout signal production. Ciliate preparedness for vibrations selectively and significantly affected the magnitude and valence of Szilárd engine performance during modal and non-modal strategy corrective cycles. But entropy fidelity for all replies mainly improved across learning trials as refinements in engine efficiency. Fidelity neared maximum levels for only modal signals coded in resilient three-bit repetition error-correction sequences. Together, these findings demonstrate microbes can elevate survival/reproductive success by learning to implement classical fault-tolerant information processing in social contexts.

## Introduction

Overcoming errors in communications and information processing is an endemic problem for all biological systems. For socially intelligent microbes capable of quite advanced cell–cell communications and cellular decision making (Ricci, 1990; Crespi, 2001; Rao et al., 2002; Winans and Bassler, 2002; Ben-Jacob et al., 2004; Nakagaki et al., 2004; Ben-Jacob, 2008; Wolf et al., 2008; Koseska et al., 2009; Clark, 2010a,b,c,d,e, 2011a,b,c, 2012a; Marijuán et al., 2010; Clark and Solé, 2013), the necessity of error-syndrome diagnosis and correction can be just as important to survive and reproduce in hostile environments as it is for phylogenetically more recent social animals (e.g., Johnston, 1995; Hauser, 1997) and “higher” plants (e.g., Trewavas, 2003). Indeed, the phenomenological similarities between microbe and animal social behaviors can be striking, suggesting a possible microbial origin for social cognition and careful use of public and private information by metazoa (e.g., Shapiro, 1998; Crespi, 2001; Winans and Bassler, 2002; Ben-Jacob et al., 2004; Michod, 2007). Fidelity of processed information may predictably determine, for instance, whether colonial prokaryotes and “lower” eukaryotes successfully communicate deceptive or honest signals to conspecifics while instigating and/or mediating conflicts that secure opportunities for improved individual or group ecological fitness. Obvious examples of such social contexts include non-clonal reproductive situations. The mating mechanisms of microbes across systematics differ considerably (Cavalier-Smith, 2002), but general commonalities in chemical signals, ostensive and non-ostensive behavioral “rituals,” and reproductive goals support the hypothesis that mate selection, itself a kind of fault inspection employed to lower the probability of genetic exchange with defective partners (Tinbergen, 1953), emerged before the evolutionary divergence of animals and fungi (Clark, 2010e, 2011a, 2012a, 2013). As with animals, microbial strategies of intra- and intermate selection advance cohort and offspring adaptation through non-promiscuous unions that minimize survival-reproductive tradeoffs (Clark, 2011a, 2012a, 2013). Making mate choices based on the perceived ecological fitness of suitors advertising superior somatic structures (e.g., ornaments and weapons), motility competence (e.g., courtship dances), social aptitude (e.g., conflict mediation and instigation), and/or other survival characteristics also promotes the vertical and, where applicable, horizontal spread of beneficial inherited traits, such as tolerance to stressors and toxins (Lujan et al., 2007), pathogen virulence (Gibson, 2001; Chandler et al., 2005; Nielsen and Heitman, 2007), biofilm formation (Ghigo, 2001), and cell aggregation (Hirt et al., 2002; Butler et al., 2009), within subdivided populations at greater rates and efficiencies (Clark, 2011a, 2012a, 2013). Although microbial mate selection appreciably favors virulence and transmission of infectious diseases, major evolutionary transitions, and emergence of primitive social intelligences (Clark, 2012a, 2013), the biological and computational processes used by microbes to identify and correct performance faults during mate selection remain, with few exceptions, poorly understood (Ricci, 1990; Clark, 2010a,b,c,d; Phadke et al., 2012).

### Computational aspects of ciliate mate selection

Microbial mate selection can be complex sequences of events (Miyake, 1981; Ricci, 1990; Clark, 2010a, 2012a, 2013), with each stage susceptible to error in intracellular information processing and/or cell–cell communication. Many ciliates, for example, detect and respond to peptide pheromones secreted by non-self mating types (e.g., Miyake, 1981; Vallesi et al., 2005). Pheromones announce the location and type of mate(s) available and, in an animal-like self- and group-referential fashion, help to identify self and kin from non-kin. Sufficiently attracted individuals may try to engage one or several compatible partners (i.e., an opposite mating type) in sexual-like conjugation normally producing eight daughter cells per reproductive pair at lifecycle's end (Lynn, 2008). In different ciliate genera, stereotypic courtship dances and dominance displays patterned with recursive movements associated with avoidance reactions (e.g., ciliary reversals and contractions) and exploration (e.g., probing behaviors and pivots) putatively effect inter- and intramate selection (Bishop, 1923; Seshachar and Padmavathi, 1959; Ricci et al., 1980; Ricci, 1990; Stock et al., 1999; Clark, 2013). These preconjugal behaviors facilitate circulation of free water-soluble mating pheromones and encourage cell–cell contacts that elicit changes in cell structure allowing partner docking and gene exchange (Seshachar and Padmavathi, 1959; Cronkite, 1975; Miyake, 1981; Ricci, 1990; Vallesi et al., 2005; Lynn, 2008; Clark, 2010a). Specific pheromone binding to extracellular domains of G-protein-associated paracrine receptors activates sexual transduction pathways which integrate somatic and reproductive processes, including cell-growth arrest, micronuclear meiotic division, zygotic nucleus formation, and maconuclear differentiation (cf. Katz, 2001). As in other eukaryotic microbes (Baneutt, 1998; Muller et al., 2003; Yoshimura et al., 2004; Lombardi et al., 2008), pheromone-activated somatic and genetic events are further modified by important partner-initiated intracellular Ca^2+^-induced Ca^2+^ cascades and phosphatase- and protein-kinase-dependent feedback regulation of mechanosensation, leading to preconjugal and conjugal ciliate motility adaptations (Miyake, 1981; Stock et al., 1999; Clark, 2010a,b,c,d,e, 2011a,c). Besides preconjugal mating displays, alterations in motility during conjugation provide additional unique benefits for ciliates not currently observed in other microbes. For instance, RNA processing of the very fragmented, macronuclear polyploid genome of ciliates enables heritable epigenetic innovations, akin to “nuptial gifts” (Clark, 2012a, 2013), to be passed from high-quality suitors to physically accessible recipients lacking motility competence (Hiwatashi et al., 1980; Pennock et al., 1988). Disparities in motility among partners jeopardize paired fitness, although a significantly inferior ciliate gains some survival and reproductive advantages for itself and its offspring upon conjugating a fitter mate. Fitter ciliates compensate for their poor mate selection via cytoplasmic exchanges that reinstate the wildtype phenotype to conjugated mutants incapable of performing programmed movements. This kind of extranuclear modification further limits fitness tradeoffs for each party, recovering typical cooperative swimming between ciliates when agile, synchronous motions and speedier escape velocities would both fend-off predation risk and improve the likelihood of finishing reproduction (Clark, 2010a, 2012a, 2013).

**Figure 1:**
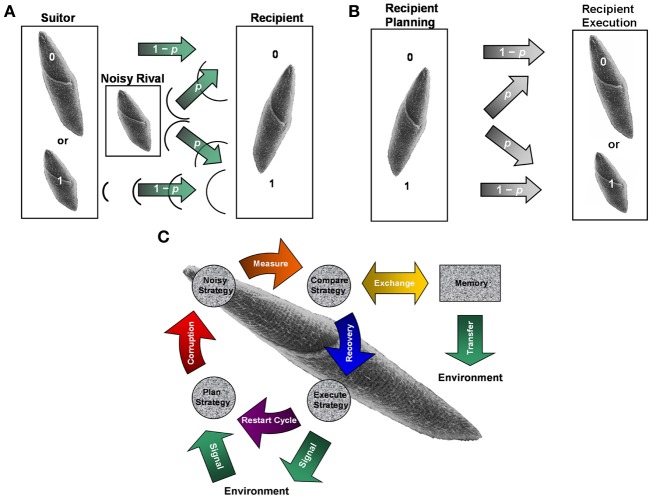
**Noisy communication channels, repetition coding, and Szilárd engine processing in ciliate signaling. (A)** Example of noisy binary symmetric bit-flip channel interfering with suitor contraction messages. An *S. ambiguum* suitor either contracts (1) or remains fully extended (0) with probability 1 − *p* of respectively sending or not sending a single courting vibration without error to a recipient *S. ambiguum*. A noisy *S. ambiguum* rival positioned between suitor and recipient emits a single contraction with a probability *p* of corrupting the suitor's message. Vibration from rival's contraction may collide with suitor's vibration, annihilating both signals and flipping the courting signal from 1 to 0. Or, vibration from rival's contraction may pass to the recipient when suitor remains fully extended, flipping the courting signal from 0 to 1. The rival's behavior is a form of social cheating, where it takes advantage of its closer physical proximity to the recipient to attract (e.g., bit flip from 0 to 1) and conjugate (e.g., bit flip from 1 to 0) a mating partner already “aroused” by the suitor's previous actions. Suitors may improve upon the reliability of single-contraction signals by sending a series of three binary responses (e.g. (1,1,1) for conspicuous consumption and (0,0,0) for prudent savings), known as repetition coding. As long as no more than one bit flip occurs in the response series, the message is transferred and decoded successfully at probability *p* < 1/2. When *p* exceeds this value, the probability of error becomes *p*_*E*_ = 3*p*^2^ − 2*p*^3^ > *p* and signal transmission loses its improved reliability over single-bit coding. Identical scenarios may occur for vibrations caused by ciliary reversals (not shown). In the present experiment, the role of suitor/rival is played by an ambiguous vibration source to create a mock-mating situation. **(B)** The concepts of noisy communication channel and repetition coding applied to recipient reply production. Once suitor signals are perceived, a recipient must plan and execute a reply potentially corrupted by intracellular noise also described by the (memory-less) binary symmetric bit-flip channel. Channel reliability for single- and three-bit coded sequences is identical to that discussed in **(A)**. **(C)** Representation of ciliate behaving as a Szilárd engine to diagnose and correct error syndromes in serial behavioral strategies. The objective of error diagnosis and correction cycles is to maintain constant Boltzmann entropy through detection of entropic deviations between strategy planning and execution stages noted in **(B)**. The engine acts as an imperfect noisy erasure channel, obeying the laws of thermodynamics by interacting with the local environment to shunt or absorb energy and/or informational equivalents of energy. Idealized erasure channels may function via Markov processes to transmit a message without error at probability 1 − *p* and to exchange a transmitted message with error at probability *p*. Thus, engine performance and error diagnosis and correction can be measured at a macroscopic scale without specific knowledge of intracellular signal transduction substrate and processes. **Panel A** adapted from Clark (2010c) with permission.

The oftentimes “programmed” or instinctual-like character of ciliate matings, similar to other eukaryotic microbes, would suggest that fault-tolerance is built into the structure and function of relatively inflexible genetic and epigenetic regulatory networks controlling signal coding, planning, execution, and decryption during preconjugal activities (cf. Cronkite, 1975; Ricci, 1990; Katz, 2001; Behar et al., 2008; Guantes et al., 2010; Lehner, 2010). But new experiments simulating mating contexts have now elaborated traditional learned aspects of ciliate intra- and intermate selection (Clark, 2010a,b,c,d,e, 2011a,b,c, 2012a, 2013), indicating non-associative and associative learning and memory processes may substantially contribute to detecting and correcting information errors, as they do in animal behavior. To facilitate mating success, the large heterotrich *Spirostomum ambiguum*, for example, learns to advertise various degrees of mating fitness to perceived “suitors” and “rivals” by serially contracting or ciliary reversing at rates that signal either conspicuous consumption or prudent savings, in accordance with the handicap principle (Clark, 2010a, 2012a; Figure 1A). Ciliates exhibiting responsiveness of conspicuous consumption declare fit reproductive status via the excessive rates at which they signal avoidance. Superior mating candidates are alone capable of metabolically wasteful displays. Since the behavior of conspicuous consumers makes it difficult for exchange of preconjugal touches between courting couples, they play “harder-to-get” when responding to the presumed advances of nearby conspecifics. Prudent savers, on the other hand, conserve energy stores for scenarios more favorable for conjugating a partner. These ciliates reply with lower frequencies of avoidance reactions, guaranteeing would-be fellow ciliates of the probability of being “easier-to-get” during courtship dances. By deciding to switch from an initial behavioral strategy that signals conspicuous consumption to one that signals prudent savings, fitter ciliates learn to altruistically sacrifice potential net payoffs to persuade suitors to participate in paired reproduction. In effect, they incur higher reproductive costs, in terms of possible offspring numbers and viability, by mating with inferior ciliates. Less fit ciliates unable to sustain long periods of high response rates may switch their initial behavioral strategy of prudent savings to briefly emit conspicuous consumption and thus learn to increase net payoffs by both opportunistically cheating finer rivals and engaging higher quality suitors in conjugal activity.

The ability of each *S. ambiguum* to appropriately stay with the same reply or to switch its reply from one behavioral strategy to another critically depends on the type of dual-process non-associative learning expressed (i.e., sensitization or habituation), the duration of learning (i.e., longer- or shorter-term), and the efficiency of heuristics formed from recursive strategy searches and use (Clark, 2010a). Heuristics represent stored patterns of action used by a ciliate. They evolve via classical Maxwell–Boltzmann, quantum Bose–Einstein, and quantum Fermi–Dirac statistics (Clark, 2010b,d) into ordered computational networks of serial escape behaviors organized around centers of smaller, local strategy groups supporting courting assurances of harder-to-get and easier-to-get (Clark, 2010a). Each node of a heuristic obeys preferential attachment rules akin to Hebbian learning rules (Hebb, 1949; Clark, 2010b,d) and contains a unique bit-string or information sequence representing perceived, planned, and executed behavioral strategies. Because heuristics encode both exemplar and frequency information about the reproductive fitness associated with mating responses, they serve as primitive representativeness and availability heuristics (cf. Clark, 2012a, 2013). Ciliate social computation networks exhibit some degree of fault tolerance through topological invariance. Furthermore, as ciliates develop their signaling skills over numerous trials, the connectivity between different strategies often strengthens from Hebbian-like learning which, in turn, promotes faster and less error-prone decisions about the quality of mating replies until a single solution is found (Clark, 2010a,b,c,d,e, 2011a,b,c, 2012a). The time taken by the best experts to master signaling decisions achieves efficiencies that resemble the quantum process of finding target solutions with Grover's search algorithm (Grover, 1996; Clark, 2010a,b,c,d,e, 2011a,b,c, 2012a). Very successful strategy searches coincide with emergence of computational analogues of Bose–Einstein condensation in signal choice (Clark, 2010b; see Bianconi and Barabási, 2001 and Bianconi, 2002a,b, 2003 for related findings involving technological networks) and of quantum tunneling in decision rates (Clark, 2010d; see Stella et al., 2005 for related research involving network search optimization via quantum annealing). These quantum computational or network phenomena approximate physical ones and are consistent with organic chemical reactions thought to produce weak Fröhlich condensation (Reimers et al., 2009) and possible quantum tunneling (McMahon, 2003) at physiological temperatures, as well as with the operation of classical and quantum mechanochemical engines that obey heat and refrigeration statements derived from the laws of thermodynamics (Hawkes and Holberton, 1975; Gore et al., 2003; Matsuno, 2006; Johal, 2009).

### Error diagnosis and correction by ciliates

Notably, the engine-like traits of intracellular bioprocesses mediating ciliate social decision making (Clark, 2010a,b,c,d) offer suitable means to limit faults and improve individual or shared ecological advantages through identification and recovery of information corrupted during noisy stages of signaling performance and message transmission, much like comparator routines implemented by artificial and hybrid technological systems (Nielsen and Chuang, 2000; Bennett, 2003; Ladyman et al., 2007). To implement error diagnosis and correction procedures, information coding schemes become necessary. Simple repetitive coding schemes are evident in animal communication to help control signal degradation of public information (cf. Hauser, 1997). Clark (2010c) first proposed the unique anticorrelated bit-string configuration of modal behavioral signals emitted by ciliates enables them to construct classical repetition (Figure 1A) and possibly superposed quantum bit-flip error-correction codes that maintain specificity of cell–cell communications as well as help prevent deterioration of mating signals from both color and white environmental noise. These sorts of classical and quantum codes contain redundant bit and qubit states which lower the probability that socially important transmitted information could be irretrievably lost to decoding. Such codes expect to be valuable in selectively conveying net reproductive fitness to upwards of hundreds of different viable mating types located in geographically constricted areas, where, for instance, isolation from conspecifics is low and likelihood of acquiring deleterious genes is high.

Moreover, single cells are themselves noisy communication channels and information coding schemes might be utilized by ciliates to process information within the cell after receiving messages and before sending replies (Figure 1B). This idea has never been well-explored for microbial signal production. Because ciliates (and other phylogenetically diverse cells) behave as physical and cybernetic engines, it is quite possible that they learn to diagnosis and correct error syndromes in the (thermal) Boltzmann entropy of codes as Szilárd-like heat and refrigeration engines (Nielsen and Chuang, 2000; Ben-Jacob, 2008; Clark, 2010a,b,c,d,e, 2011b,c Figure 1C). The present study (Box 1) examined these specific questions for the first time under mating game conditions by assessing whether or not ciliates of differential mating responsiveness: (a) use *classical* repetition codes to help noise-protect serial behavioral strategies during signal production, and (b) learn to change fidelity between strategy planning and execution as a consequence of modifications in *classical* engine performance with each simulated perimating experience. New analyses of data collected from prior experiments designed to evaluate other aspects of ciliate learning (cf. Hamilton et al., 1974; Clark, 2010a,b) showed coding schemes prevented all executed behavioral strategies from exceeding bit-flip error thresholds. In addition, ciliates, consistent with Ca^2+^-dependent Hebbian-like comparator learning models, were found to detect deviations in Boltzmann entropy content of mating replies passed between signal planning and execution stages over iterative mock social trials. Recovery or maintenance of coded states, constituting a classical Markov process, occurred when lowering or rising entropy drove respective “refrigeration” and “power” strokes from the planning information reservoir to the execution information reservoir, where entropy associated with prior stored codes served as reference for error checking and was then dissipated or extracted upon signal execution. Fidelity between the two reservoirs improved for all mating signals, but near perfect fidelity was often learned for only modal error-correction codes by the end of testing. (See Box 2 for glossary of terminology).

Box 1 **Highlights.**

- Ciliates learn repetition coding to safeguard mating signals from ambient noise.
- We find same codes help ciliates diagnose and correct errors in signal production.
- Repetition codes prevent executed signals from exceeding bit-flip error thresholds.
- Ciliates act as Szilárd engines to improve fidelity of signal planning/execution.
- Modified engine performance increases mating competency via learned Markov process.

Box 2 **Glossary of terminology.**

**Bose-Einstein Statistics**

Statistics independently created by Satyendra Bose and Albert Einstein that predict the unrestricted distribution of a quantum mechanical system containing indistinguishable, non-interacting particles over energy ground states or quanta in thermal equilibrium. Such systems only allow two polarization states of integer spins for particles, called bosons, with identical wave functions. The thermodynamic limit, caused by critical ultralow temperatures, brings the system to a coherent or superposed macroscopic quantum state known as Bose-Einstein condensation. An analogue of Bose-Einstein condensation can occur in the operation and organization of complex technological and biological networks which converge onto one computational state. The connectivity of these quantum networks obey Bose-Einstein statistics when nodal strength is described as separate fitness or energy levels and nodal links take on the identity of particle states functioning under associative-like preferential attachment rules. In such cases, control parameter *T* (i.e., local absolute temperature), which dictates system behavior, is often replaced with a computational annealing parameter, such as space, time, or the “critical tunneling field strength.” The rate of state transitions or computational decisions in a quantum network also follows non-linear first-order Arrhenius kinetics associated with quantum tunneling, making it a computational or network analogue of the physical phenomenon.

**Handicap Principle**

A consumption-value argument of fitness originated by Amotz Zahavi, the handicap principle advocates honest signaling evolves along some measurable dimension, such as ornate or modest morphology or behavior, which incurs ecological cost for communicants capable of deception. The fittest communicants, known as conspicuous consumers, express their superiority to observers by flaunting their traits in disregard of potential risks, including predation, metabolic stress, and injury. In contrast, inferior communicants, known as prudent consumers, are less likely of surviving such risky, extravagant displays and emit frugal or careful signals.

**Hamming Metrics**

Static measure of the relatedness or difference between two bit strings or ‘words’ devised by Richard Hamming. The Hamming distance quantifies the number of bit places that differ between two bit strings of equal length. Whereas, the Hamming weight quantifies the number of bit places that differ between a bit string and an idealized bit string containing all zeros. Both of these values can be converted to other units, such as entropies of different degrees of freedom, via Landauer's principle.

**Hebbian Learning**

An iterative adaptive control mechanism utilizing activity-dependent bidirectional or dual-processes associative learning rules to either strengthen or weaken nodal connections of an associative (biological or technological) network. The set of learning rules (e.g., cooperativity, coactivity or associativity, synaptic or nodal efficacy/weight, etc.) governing this kind of feedback regulation, whether at neuronal synaptic junctions or other nodal forms of computational circuitry, are named after Donald Hebb, who is largely acknowledged as the founder of such concepts.

**Fermi-Dirac Statistics**

Statistics independently created by Enrico Fermi and Paul Dirac that describe the distribution of indistinguishable, non-interacting particles of a quantum mechanical system in thermal equilibrium over single-particle energy levels. Such systems only allow polarization states of half-integer spins for particles, called fermions, with identical wave functions. Particles obey the Pauli exclusion principle, which states no two identical particles can occupy the same energy level. Similar to Bose-Einstein statistics, Fermi-Dirac statistics describe the connectivity and behavior of some technological and biological networks, where again nodal strength forms separate fitness or energy levels and nodal links become particle states operating under associative-like preferential attachment rules. These quantum networks, like their Bose-Einstein counterparts, produce statistical phenomena, such as computational tunneling effects, analogous to quantum mechanical effects observed for physical systems.

**Grover's Search Algorithm**

A member of the class (or family) of procedures that implement some or all of the properties of quantum mechanics to enhance information processing capacity and speed over that achieved by standard classical procedures. Discovered by Lov Grover, Grover's algorithm relies on an initial quantum superposition of arbitrary *N* eigenstates to later execute non-classical “subroutines” involving unitary phase shifts on eigenstates and to produce a root-rate gain in the algorithmic time [i.e., O(*N*^1/2^)] needed to arrive at some “target” variable *x*.

**Markov Process**

The process of change, such as with information content, in a system characterized by the Markov property, named after the mathematician Andrey Markov, that future states are independent of past states given the present state (a first-order Markov process).

**Maxwell-Boltzmann Statistics**

Named after its creators, James Clerk Maxwell and Ludwig Boltzmann, Maxwell-Boltzmann statistics describe the distribution of distinguishable classical particles in thermal equilibrium ranges with negligible quantum effects. As with quantum networks and their statistical mechanical properties, Maxwell-Boltzmann statistical properties emerge from the connectivity and behavior of technological and biological networks operating in classical computational phases. For example, classical networks obeying associative-like preferential attachment rules show linear first-order Arrhenius kinetics in the rate of state transitions or computational decisions. Depending on functional characteristics involving control parameters, a network may switch between computational phases dominated by classical or quantum regimes.

**Memory Channels**

A broad class of communication channels, classical memory channels, unlike binary symmetric channels, can store as well as conduct noiseless or noisy binary signals. A special subclass of memory channels, termed erasure channels (e.g., binary erasure channels and packet erasure channels), diagnose and correct corrupted information by replacing it with error-free codes stored in a memory register. Output of perfect or ideal memory channels is errorless.

**Non-associative Learning**

Divided into sensitization, an exponential response increment to a repeated stimulus, and habituation, an exponential response decrement to a repeated stimulus, this form of learning may be expressed in long- and short-term durations. There are well defined criteria (e.g., failure to form associations, stimulus specificity and generalization, learning performance inversely related to stimulus intensity, dissensitization/dishabituation, etc.) for establishing non-associative learning at cellular, tissue, systems, and organismal levels of biological organization under the dual-process model. Although the biomechanisms of learning may differ within and across phylogenetic categories, both sensitization and habituation are observed for microbes and animals.

**Power and Refrigeration Cycles**

Cycles or strokes of respective heat and refrigeration engines used to perform measurable amounts of work. The effectiveness of engine performance can be quantified with thermodynamic sensitive indices of efficiency (heat engine) and coefficient of performance (refrigeration engine). For information channels, engine work performs computations, such as information transmission and error diagnosis and correction. Power cycles correspond to entropic information transfer associated with memory erasure, whereas refrigeration cycles correspond to negentropic information transfer associated with memory storage.

**Quantum Bit-Flip Coding**

This code belongs to a family of codes generally termed quantum error-correction coding that enlists quantum computation and information theory to safeguard information against noise. In quantum information theory, the classical bit is replaced with the quantum bit or qubit. A single qubit is the orthonornal unit vectors or basis states |0 〉 and |1 〉 for a two dimensional vector space put into linear combination or superposition: |ψ 〉 = α |0 〉 + β |1 〉, where variables α and β are complex numbers called vector amplitudes. Measuring a qubit after communication transmission yields a single pure state of 0 or 1 with respective probabilities |α |^2^ and |β |^2^, so that |α |^2^ + |β |^2^ = 1. The quantum bit-flip code, represented as superposed logical coding scheme of three qubits, |ψ 〉_*L*_ = α |000 〉 + β |111 〉, upholds the no-cloning theorem and has respective probabilities *p* and 1 − *p* of being transmitted through a noisy quantum bit-flip channel with and without error.

**Repetition Coding**

This simple classical error-correction code contains redundant information in a bit string of three or more bits. If the coded string becomes corrupted, the redundant information maintains a high probability of being retrieved from the original message or reply in a process called “majority voting” decoding. This type of error-correction only works for classical digital information passing through a noisy communication channel, such as the binary symmetric channel having normalized probabilities *p* > 0 and 1 − *p* > 0 of transmitting information with and without error, respectively.

**Representativeness and Availability Heuristics**

Simple intuitive logic systems used by microbes and animals famously studied in humans by Amos Tversky and Daniel Kahneman. Representativeness heuristics reduce inferential tasks to quick exemplar or similarity comparisons. When traits or events used for judgments are unrepresentative or provide only indefinite dimensions for categorization, such as level of mating fitness and availability, the heuristic may become a non-normative guide to decision making. Availability heuristics employ readily accessible information to judge the frequency, probability, and causality of traits and events. Because decisions result from the accessibility of perceived and/or retrieved information, these simple rules of thumb may be corrupted by inaccurate trait or event base rates under the control of subjective factors. Under such conditions, like representativeness heuristics, availability heuristics become a non-normative guide to decision making.

**Szilárd Engine**

Expanding the concept of Maxwell's demon, Leó Szilárd developed the idea of an engine capable of manipulating information as a physical engine with partitioned heat or refrigeration reservoirs containing excitable particles. Selective motion or cycling of particles from one reservoir to the other, measured by the demon, lowers the system's entropy (i.e, negentropy) in apparent violation of heat and refrigeration statements of the second law of thermodynamics. However, the missing entropy is stored in the demon's finite-capacity memory, where information must be eventually erased and transferred to the environment to store new information. Information erasure reinstates or increases the total entropy of the combined system (engine and demon), thereby complying with the laws of thermodynamics by way of Landauer's principle.

## Materials and methods

### *Spirostomum ambiguum* culture, stimulation, and behavior

*S*. *ambiguum* were purchased from Connecticut Valley Biological Supply Company, Southhampton, MA and cultured as reported elsewhere (Hamilton et al., 1974; Clark, 2010a,b). Large, free-swimming protozoa were selected, placed in a round sterile slide well-containing culture medium, and then covered by a moist gasket ring and evaporation coverlet for individual study (Hamilton et al., 1974; Clark, 2010a,b). *S*. *ambiguum* behavior and slide well conditions were allowed to stabilize at room temperature for 10 min (Osborn et al., 1973) on the stage of a Nikon M inverted microscope. Next, a percussion cylinder or piston, producing a 0.0011-N force and driven by constant spring action (*k*_*S*_ = 0.1158) at a rate of 0.1 Hz for 10 min, was delivered to the slide surface outside the well perimeter (Hamilton et al., 1974; Clark, 2010a,b). The piston upon impact created a vibration in the form of a longitudinal shock wave that traveled through the culture medium. A total of 60 stimuli were administered to each protozoan tested. No ciliate participated in more than one experiment.

Two behaviors of *S. ambiguum* used during mating situations were of interest for this study (Clark, 2010a). Contractions, a rapid vibration-evoked shortening to about half resting length, and ciliary reversals, a vibration-evoked movement of 0.3 mm or greater in the posterior direction, were viewed by dark-field microscopy at no more than 10× magnification and recorded onto analog video tape for later analyses (Hamilton et al., 1974). Two independent raters classified contractions and reversals by double-blind techniques (Hamilton et al., 1974). Data for *S. ambiguum* that developed abnormal body shapes, swimming activity, or spontaneous contractions during experimentation were discarded. Initial responsiveness to vibrations, Ω_Initial_, was determined from the frequency or probability of making responses to the first five stimuli delivered (Hamilton et al., 1974; Hamilton, 1975; Eisenstein et al., 1982; Eisenstein and Eisenstein, 2006; Clark, 2010a,b). Protozoa were separated into one of three intervals of initial responsiveness, 0.0–0.2, 0.4–0.6, and 0.8–1.0. These probabilities corresponded to response rate intervals of 0.00–0.02 Hz, of 0.04–0.06 Hz, and of 0.08–0.1 Hz for respective initial low (*n* = 14), medium (*n* = 16), and high (*n* = 10) contraction responsiveness and initial medium (*n* = 17) and high (*n* = 23) reversal responsiveness groups. All ciliates were classified into one of three contraction responsiveness groups and one of two reversal responsiveness groups. No ciliate displayed initial low reversal responsiveness. Additionally, microbes rarely showed the same level of responsiveness for contractions and reversals. Initial responsiveness for these same ciliates has been previously demonstrated to correlate with the expression of different types of non-associative contraction and reversal learning (Hamilton et al., 1974; Hamilton, 1975; Eisenstein et al., 1982; Eisenstein and Eisenstein, 2006; Clark, 2010a), of serial contraction and reversal strategies signaling different levels of mating fitness (Clark, 2010a,b,c,d), of heuristics used to make decisions about social commitments (Clark, 2010a,b,c,d, 2012a), and of classical and quantum-level performance characteristics (Clark, 2010a,b,c,d,e, 2011b,c, 2012a). Initial behavioral responsiveness was used here for the first time to identify possible differences in the way ciliates detect and correct information processing errors during the planning and execution of mating strategies.

### Computational characteristics of serial mating strategies and strategy state spaces used to make mating decisions

*S. ambiguum* uses a set or repertoire of classical serial contraction strategies, *C*, and serial reversal strategies, *R*, of differential responsiveness Ω when responding to iterative vibrations (Clark, 2010a,b,c,d; see Appendix). For every set of executed serial contraction strategies, *C*_*E*_, or reversal strategies, *R*_*E*_, there is a corresponding set of planned states, *C*_*P*_ and *R*_*P*_. These sets were defined here as one-to-one onto state space mappings or topological conjugates with the following traits. State space structures emerge from both local and global patterns of activity exhibited by a state or a set of states located within a defined computational *n*D space. Trajectories through state spaces correspond to behavioral heuristics or rules for patterns of action attracting ciliates into certain contraction or ciliary reversal dynamics signaling mating fitness (Clark, 2010a,b). All-or-none binary sequencing of each stimulus-evoked contraction or reversal event, where zero equaled no response and one equaled response, produced the computational basis for classical 3D behavioral state spaces and conjugate spaces, *C*_*E*_ and *C*_*P*_ and *R*_*E*_ and *R*_*P*_ (Clark, 2010a). Basis strings spanned entire test sessions, forming a time series of {*s*_*n*_|*n* = 1, …, 60} for contractions and for reversals due to *n* vibrations. Resulting basis strings were subsequently transformed into states of ordered triplets, the Cartesian coordinates of 3D state spaces, via the sequential-series method (Eagan and Partridge, 1989). 3D classical state spaces for executed contraction and reversal behaviors took the metrical form:

(1) $$S_{m\times d}=\left[\begin{array}{l} s1,s2,\ldots ,sd\\ s2,s3,\ldots ,sd+1\\ \vdots \\ sm,sm+1,\ldots ,sn \end{array}\right],$$

where *m* is the length of the basis string of contractions and *d* is the embedding dimension. Planned behaviors formed a conjugate classical space to S_*m × d*_. This simple method has proven as reliable and valid as other methods that employ phase relationships for classifying self-referential non-linear dynamics of observable temporal events (Kelso, 1995; Yunfan et al., 1998), with particular success in classifying evoked patterns of neuronal discharge (Eagan and Partridge, 1989; Ryan, 2000). Futhermore, Takens' embedding theorem (Takens, 1981) importantly proves that if the number of dimensions of the reconstructed system (e.g., 3D state space) is as large as 2*n* + 1, where *n* is the dimension of the original system (e.g., 1D basis string or sequential series), then a one-to-one smooth mapping between the reconstructed system and the original one exists.

A total of 2^*D*^ possible executed strategies, *c*_*E*_ and *r*_*E*_, and planned states, *c*_*P*_ and *r*_*P*_, of three consecutive responses, where base equaled binary response basis and exponent equaled number of state space dimensions, could be embedded within respective *C*_*E*_ and *C*_*P*_ and *R*_*E*_ and *R*_*P*_ spaces (cf. Clark, 2010a). Each of the eight possible 3D state space coordinates, (0,0,0), (0,0,1), (0,1,0), (1,0,0), (0,1,1), (1,1,0), (1,0,1), and (1,1,1), trivially corresponded to one unique first-order executed or planned response strategy with a value for responsiveness, Ω, as discussed in the preceding subsection (see Appendix for more details) and with an upper limit of 58 possible occurrences over 60 stimulus trials. These behavioral strategies conform to classically encoded repetition signals which safeguard messages and replies form information loss and corruption (Clark, 2010c). Upper limits on strategy occurrences were determined from the number of stimuli presented, from responses evoked, and from computational space dimensions, by way of the sequential series method. The most repeated strategy used by each protozoan over the course of testing was designated a modal serial strategy. A modal strategy corresponds to an over-learned fit strategy, by preferential attachment rule standards (Clark, 2010b). Non-modal serial strategies were all other strategies. They correspond to under-learned, unfit strategies, by preferential attachment rule standards (Clark, 2010b). The period of any one individual state was constantly 30 s and was the summation of three consecutive interstimulus (i.e., intervibration) intervals. Strategies (0,0,0) and (1,1,1) were located at opposite ends of the response continuum, with (0,0,0) being ideal low responsiveness (i.e., ideal prudent savings) and (1,1,1) being ideal high responsiveness (i.e., ideal conspicuous consumption). Moreover, different combinations and sequences of serial behavioral strategies from the above set of 2^*D*^ strategy permutations limit respective state spaces with *n* vertices to a manifold formed from non-simply connected paths (Clark, 2010a). Elementary groups of different homology corresponded to path connected or grouped strategies contained within a topologically invariant computational network. Thus, the trajectories connecting strategies represent the sequence of transitions or computations (i.e., decisions) made during strategy planning and execution.

### Boltzmann entropy, the generalized work-energy theorem, and Szilárd's engine for diagnosing and correcting error syndromes in serial mating strategies

Diagnosis and correction of information errors by ciliates may be considered a sort of thermodynamic process following equivalent heat-engine and refrigerator statements derived from the second law of thermodynamics (Nielsen and Chuang, 2000; Bennett, 2003; Ladyman et al., 2007). These statements affirm that an engine/refrigerator performing cycles cannot produce another effect than transferring heat from a hot/cold reservoir to a cold/hot reservoir with which an equal amount of work is accomplished. Therefore, the objective of ciliates, if they perform error-syndrome diagnosis and correction between strategy planning and execution stages, is to maintain entropy content by extracting/rejecting deviating entropy associated with errors in internally transmitted and stored information over cycles of signal production (Figure 1C). This is a Szilárd engine interpretation of the classical processes performed by Maxwell's demon, which filters syndrome measurements then corrects errors based on comparisons with stored information. The second law of thermodynamics is upheld when stored measurements are “erased” or “reset” in accordance with Landauer's principle for memory devices of finite storage capacity (Clark, 2010b,d,e).

To evaluate ciliates' error diagnosis and correction capacity and to be consistent with previous analyses (Clark, 2010b,d,e) and with Shannon's noisy channel coding theorem for Markov processes over an erasure channel (Nielsen and Chuang, 2000), the entropy content of planned and executed serial behavioral strategies was calculated as respective expected (planned) and observed (executed) Boltzmann entropies, a convenient and valid index of engine performance in (thermal) physical degrees of freedom. Four sets of Shannon entropy were first determined for each ciliate tested. Two of these entropies, known as the entropy rate of the source (i.e., intracellularly encoded signal of planning stage; cf. Nielsen and Chuang, 2000), were defined as Shannon's entropy *H*(*Y*_*n*_) = − *p_y_* log_2_ *p_y_* and *H*(*U_n_*) = − *p*_*u*_log_2_*p*_*u*_, where *y*≤ *c*_*P*_ and *u* ≤ *r*_*P*_ denote a unique planned strategy over a range of 5 ≤ *y*, *u* ≤ 8 strategies. Thus, entropies were calculated for each respective serial contraction, *c*_*P*_, and reversal, *r*_*P*_, strategy planned by a unique ciliate for each sequential interval associated with a *s*_*n*_ learning trial. Bayesian probabilities *p*_*y*_ and *p*_*u*_ for each strategy fell within the respective Bayesian probability distributions Ψ_*P*_ = {*Y*_*n*_|*n* = 1, …, 58} and Φ_*P*_ = {*U*_*n*_| *n* = 1, …, 58} of respective grand *Y*_*n*_ × 2^*D*^ and *U*_*n*_ × 2^*D*^ matrices containing the expected probabilities for the occurrences of subsets of planned modal and non-modal serial contraction and reversal strategies within respective contraction and reversal conjugate spaces *S*_*m × d*_. Thus, *p*_*y*_ and *p*_*u*_ = *p*_0_ = (*x*_1_*x*_2_*x*_3_), where *p*_0_ is the expected probability of occurrence for a particular serial behavioral strategy occurring during a particular *s*_*n*_ learning trial and *x* is the expected probability for the occurrence of a response or a no response at different sequence positions of a particular serial behavioral strategy. The remaining two entropies, known as the entropy rate of the receiver (i.e., intracellularly decoded signal of execution stage), were defined as Shannon's entropy *H*(*Z*_*n*_) = − *p*_*z*_log_2_*p*_*z*_ and *H*(*V*_*n*_) = −*p*_*v*_log_2_*p*_*v*_, where *z* = *c*_*E*_ and *v* = *r*_*E*_ denote a unique strategy over a range of 5 = *z*, *v* = 8 strategies. Thus, entropies were calculated for each respective serial contraction, *c*_*E*_, and reversal, *r*_*E*_, strategy executed by a unique ciliate for each sequential interval of each *s*_*n*_ learning trial. Bayesian probabilities *p*_*z*_ and *p*_*v*_ for each strategy fell within the respective Bayesian probability distributions Ψ_*E*_ = {*Z*_*n*_| *n* = 1, …, 58} and Φ_*E*_ = {*V*_*n*_| *n* = 1, …, 58} of respective grand *Z*_*n*_ × 2^*D*^ and *V*_*n*_ × 2^*D*^ matrices containing the observed probabilities for the occurrences of subsets of executed modal and non-modal serial contraction and reversal strategies within respective contraction and reversal spaces *S*_*m × d*_. In addition, the capacity, *C*_*R*_, for a noisy erasure channel, *N*_*R*_, for contraction computations was defined as *C*_*R*_(*N*_*R*_) = *H*(*Y*_*n*_:*Z*_*n*_) and the capacity, *C*_*R*_, for a noisy erasure channel, *N*_*R*_, for reversal computations was defined as *C*_*R*_(*N*_*R*_) = *H*(*U*_*n*_:*V*_*n*_). The maximum capacity of each channel was taken over the respective source distributions of the expected probabilities of occurrence for planned strategies and is often greater than that of noisy binary symmetric channels because ideal erasure channels assume zero error following erasure and signal replacement (Nielsen and Chuang, 2000).

All of these concave entropy measures obey the generalized work-energy theorem, *W* = Δ*E* (Tipler, 1991), when *H*(*Y*), *H*(*Z*), *H*(*U*), and *H*(*V*) are converted to energy via Landauer's principle, *E* = *Hk_*B*_T*ln2 (Nielsen and Chuang, 2000), where *H*(·) is an arbitrary Shannon entropy, *k*_*B*_ is Boltzmann's constant and *T* is culture temperature in degrees K. Boltzmann entropy, *S*, was calculated from the preceding formula without inclusion of the variable *T* and was substituted for *E* into the following equations for work, power, efficiency and coefficient of performance. Work, *E*_planned_ − |*E*_executed_|, quantified the amount of incurred error as well as the amount of Boltzmann entropy extracted and/or rejected to perform error syndrome diagnosis and correction with power *P* = Δ*W*/Δ*t*, (heat engine) efficiency ε = *W/E*_planned_, and (refrigeration engine or heat pump) coefficient of performance COP = *E*_executed_/*W*. Negative values of work, power, and efficiency signify activity of a refrigeration engine or heat pump. The effectiveness of error detection and correction of information processed between strategy planning and execution stages was measured using fidelity, *F*(*p*_*P*_, *p*_*E*_) = (*p*_*P*_*p*_*E*_)^1/2^, where *p*_*P*_ and *p*_*E*_ are the respective Bayesian probabilities for planned (i.e., *p*_*y*_ and *p*_*u*_) and executed (i.e., *p*_*z*_ and *p*_*v*_) behavioral strategies. Note that these comparisons did not include planned serial behavioral strategies with introduced bit-flip errors allowing determination of error thresholds (see below subsection). Instead, remaining levels of error were revealed by work levels and less than perfect fidelity between the (expected) probability of planning a serial behavioral strategy of responsiveness Ω and the (observed) probability of executing the same serial strategy of responsiveness Ω during learning state *s*_*n*_.

### Error-thresholds for the repetition codes of serial mating strategies introduced with bit-flip errors

In addition to energy-dependent processes, ciliates may protect intracellularly transmitted information with different coding schemes (Clark, 2010c; Figure 1B). Repetition coding is a simple error-correction technique that incorporates redundant key information in a coded bit string. If part of the coded string becomes corrupted, the key information maintains a high probability of being retrievable from the original message or reply in a process called “majority voting” decoding. This type of error-correction only works for classical digital information passing through a noisy communication channel, such as the binary symmetric channel or the presently reported erasure channel having normalized probabilities *p* > 0 and 1 − *p* > 0 of transmitting information with and without error, respectively (Nielsen and Chuang, 2000). Repetition coding is evident in heuristic-guided social commitments advertised by ciliates (Clark, 2010c; Figure 1A). Sequences of all-or-none contractions and all-or-none ciliary reversals are stored by ciliates as three-bit signaling strategies in separate topologically invariant computational networks. Strategy (0,0,0), signaling ideal prudent savings, and strategy (1,1,1), signaling ideal conspicuous consumption, appear to function as orthonormal logical 0 and logical 1 codes with additional copies of unit basis states 0 or 1 completing the bit string. Bit copies permit an incipient message or reply of 0 (i.e., no response), signaling prudent savings, or 1 (i.e., response), signaling conspicuous consumption, to be transmitted between strategy planning and executed stages of signal production with a bit-flip error corrupting the code. As long as no more than one bit flip occurs in the sequence of three contractions or ciliary reversals, the message or reply is transferred and decoded successfully at probability *p* < 1/2. When *p* exceeds this value, the probability of error becomes *p*_*E*_ = 3*p*^2^ − 2*p*^3^> *p* and signal transmission loses its improved reliability over single-bit coding. To determine whether ciliates exploit the improved reliability of repetition coding for intracellular processing of mating signals (Figure 1B), separate bit-flip errors were mathematically introduced at each bit location in each three-bit behavioral strategy planned by each ciliate during a *s*_*n*_ learning trial. Errors then were averaged across the signal sequence. Thus, estimated Boltzmann entropy of each error-introduced strategy planned by a ciliate for each of *s*_1_ … *s*_*n*_ trials was calculated according to the following formula: *S*_*s*_*n*__ = ((*H(f*_1_, *x*_2_, *x*_3_) + *H(x*_1_, *f*_2_, *x*_3_) + *H(x*_1_, *x*_2_, *f*_3_))/3)*k*_*B*_ln2, where *H*(*x*_1_, *x*_2_, *x*_3_) is the Shannon entropy derived from the expected probability of occurrence, *p*_0_ = (*x*_1_*x*_2_*x*_3_), for a particular planned serial behavioral strategy, *x* is again the expected probability of occurrence for a single response (or no response) at a particular location in that planned serial strategy, and *f* is the expected probability of a bit flip at a particular location in that planned serial strategy. *S*_*s*_*n*__, therefore, is proportional to the Hamming distance from the initial errorless planned strategy state of *H*(·) and *S*(·) (Nielsen and Chuang, 2000). Each error-threshold Boltzmann entropy was then compared with the observed Boltzmann entropy of the corresponding executed serial behavioral strategy. If repetition coding succeeded in safeguarding intracellularly processed information from corruption, then real errors incurred in executed serial behavioral strategies should be statistically less or no different than the bit-flip error-threshold values introduced in planned strategies. That is, the entropic distance of an executed strategy from the corresponding errorless planned strategy should be less or no different than the entropic distance of a planned strategy with averaged bit-flip errors from the corresponding errorless planned strategy.

## Results and discussion

### Ciliate preparedness determined some indices of Szilárd engine performance during mating signal production

Ciliate preparedness, as calculated by behavioral responsiveness, a probabilistic homeostatic or internal disposition of a ciliate, such as being “alerted” or “relaxed” (Hamilton et al., 1974; Eisenstein and Eisenstein, 2006; Clark, 2010a) determines how a protozoan perceives and reacts to the contents of its surrounding environment (Clark, 2010a, 2012a). For example, protozoa beginning testing at low or high behavioral responsiveness have a propensity to perceive messages sent from the ambiguous vibration source, serving as presumptive conjugal suitors or rivals, as containing different meanings even though those messages may have the same information content (Clark, 2010a, 2012a). The same protozoa then tend to learn to plan and execute replies of different meaning based on these social biases, using modal serial strategies of ideal prudent savings or conspicuous consumption, respectively advertising polar easier-to-get or harder-to-get levels of mating commitment. Furthermore, signals of ideal prudent savings and conspicuous consumption have greater mating significance in terms of net reproductive payoffs when compared to alternative mating replies (Clark, 2010a). Because preparedness impacts the type of serial behavioral signals generated and executed by ciliates and, therefore, the degree of mating success (Clark, 2010a, 2012a,b), it seems likely that preparedness also should dictate the quality or quantity of errors incurred during signal production as well as how those errors are diagnosed and corrected before transmission of mating replies. Collapsed across learning trials and across modal and non-modal strategies, a 3 × 5 repeated-measures factorial analysis showed level of initial contraction responsiveness of microbes only produced weakly significant overall interaction effects with Szilárd-engine operational characteristics, including mean average work, efficiency, power, coefficient of performance, and fidelity associated with strategy planning-execution corrective cycles [*F*_(8, 855)_ = 1.908, *p* = 0.056]. Partial contrasts across learning trials and strategy types established that this interaction resulted from significant differences between initial contraction responsiveness groups and mean average fidelity between strategy planning and execution stages [*F*_(2, 171)_ = 19.07, *p* = 3.33 × 10^−8^; $\bar{x}$_*L*_ = 0.285 SEM = 0.010, $\bar{x}$_*M*_ = 0.198 SEM = 0.009, $\bar{x}$_*H*_ = 0.179 SEM = 0.011]. No significant effects for comparable overall two-factor repeated measures analysis of initial reversal responsiveness and Szilárd-engine operational characteristics were found. However, like initial contraction responsiveness, planned single-factor simple comparisons across learning trials and strategy types yielded significant differences for levels of reversal responsiveness and mean average fidelity between strategy planning and execution stages [*F*_(1, 114)_ = 16.126, *p* = 1.07 × 10^−4^; $\bar{x}$_*M*_ = 0.240 SEM = 0.009, $\bar{x}$_*H*_ = 0.308 SEM = 0.009]. Thus, a ciliate's preparedness influenced the magnitude of fidelity achieved for information being processed at different stages of signaling strategy production.

### Ciliate preparedness determined success of repetition codes to noise-protect contraction mating signals

**Figure 2:**
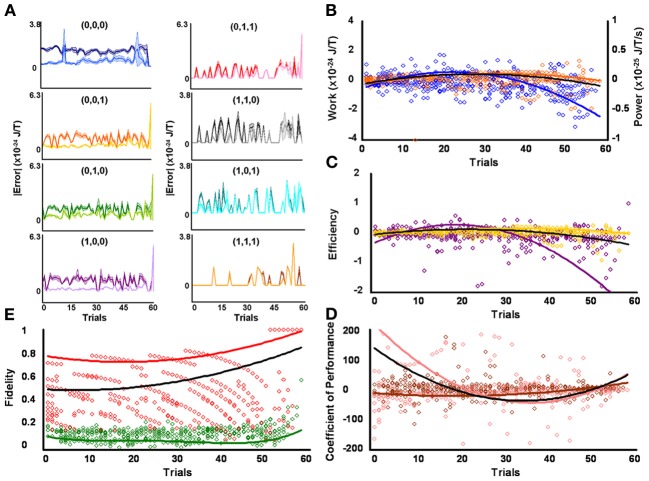
**Initial low responders learn to diagnose and correct classical errors in executed contraction strategies signaling mating fitness. (A)** Repetition coding prevented errors in executed serial contraction signals from exceeding estimated single bit-flip errors in absolute mean average Boltzmann entropy [executed (light blue) and bit-flipped (dark blue) strategy (0,0,0), executed (light orange) and bit-flipped (dark orange) strategy (0,0,1), executed (lime green) and bit-flipped (green) strategy (0,1,0), executed (lavender) and bit-flipped (purple) strategy (1,0,0), executed (pink) and bit-flipped (red) strategy (0,1,1), executed (gray) and bit-flipped (black) strategy (1,1,0), executed (turquoise) and bit-flipped (teal) strategy (1,0,1), executed (gold) and bit-flipped (brown) strategy (1,1,1)]. **(B)** Differences in mean average Boltzmann entropy between strategy planning and execution stages were expelled by ciliates when learning to detect and correct errors in modal (orange) and non-modal (blue) serial contraction signals. Negative trends in work and associated power functions show that serial strategies underwent refrigeration cycles of increasingly greater magnitude to maintain constant entropy levels. **(C,D)** Trends in mean average efficiencies and coefficients of performance for modal (gold and pink, respectively) and non-modal (purple and brown, respectively) serial contraction signals corresponded with work and power functions, confirming that ciliates behave like Szilárd engines employing refrigeration cycles when necessary to correct signaling strategy errors. **(E)** Higher trends in the mean average fidelity of Boltzmann entropy between strategy planning and execution stages indicate repetition coding was more successful in protecting modal (red) than non-modal (green) serial contraction strategies from error. Ciliates learn via Markov Szilárd-engine processes to improve fidelity between strategy planning and execution stages for all strategies for each corrective cycle. All trend lines for panels **(B)** through **(E)** are fitted to second-order polynomial equations. Black trend lines represent composites of modal and non-modal serial strategy trends. Symbol T in panels **(A)** and **(B)** is temperature in °K.

**Figure 3:**
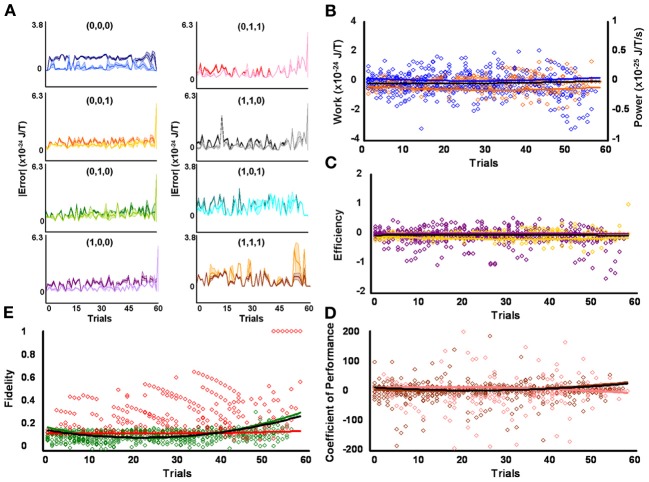
**Initial medium responders learn to diagnose and correct classical errors in executed contraction strategies signaling mating fitness. (A)** Repetition coding prevented errors in executed serial contraction signals from exceeding estimated single bit-flip errors in absolute mean average Boltzmann entropy [executed (light blue) and bit-flipped (dark blue) strategy (0,0,0), executed (light orange) and bit-flipped (dark orange) strategy (0,0,1), executed (lime green) and bit-flipped (green) strategy (0,1,0), executed (lavender) and bit-flipped (purple) strategy (1,0,0), executed (pink) and bit-flipped (red) strategy (0,1,1), executed (gray) and bit-flipped (black) strategy (1,1,0), executed (turquoise) and bit-flipped (teal) strategy (1,0,1), executed (gold) and bit-flipped (brown) strategy (1,1,1)]. **(B)** Modest differences in mean average Boltzmann entropy between strategy planning and execution stages were expelled by ciliates when learning to detect and correct errors in modal (orange) and non-modal (blue) serial contraction signals. Modest negative trends in work and associated power functions show that serial strategies experienced refrigeration cycles of increasing magnitude to maintain constant entropy levels. **(C,D)** Trends in mean average efficiencies and coefficients of performance for modal (gold and pink, respectively) and non-modal (purple and brown, respectively) serial contraction signals corresponded with work and power functions. **(E)** Trends in the mean average fidelity of Boltzmann entropy between strategy planning and execution stages indicate repetition coding was equally successful in protecting modal (red) and non-modal (green) serial contraction strategies from error. Ciliates learn via Markov Szilárd-engine processes to improve fidelity between strategy planning and execution stages for all strategies with each corrective cycle. All trend lines for panels **(B)** through **(E)** are fitted to second-order polynomial equations. Black trend lines represent composites of modal and non-modal serial strategy trends. Symbol T in panels **(A)** and **(B)** is temperature in °K.

**Figure 4:**
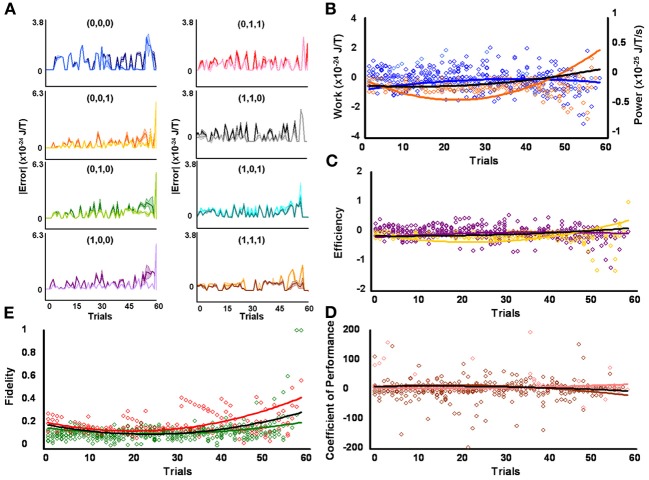
**Initial high responders learn to diagnose and correct classical errors in executed contraction strategies signaling mating fitness. (A)** Repetition coding prevented errors in executed serial contraction signals from exceeding estimated single bit-flip errors in absolute mean average Boltzmann entropy [executed (light blue) and bit-flipped (dark blue) strategy (0,0,0), executed (light orange) and bit-flipped (dark orange) strategy (0,0,1), executed (lime green) and bit-flipped (green) strategy (0,1,0), executed (lavender) and bit-flipped (purple) strategy (1,0,0), executed (pink) and bit-flipped (red) strategy (0,1,1), executed (gray) and bit-flipped (black) strategy, executed (turquoise) and bit-flipped (teal) strategy (1,0,1), executed (gold) and bit-flipped (brown) strategy (1,1,1)]. **(B)** Differences in mean average Boltzmann entropy between strategy planning and execution stages were expelled by ciliates when learning to detect and correct errors in modal (orange) and non-modal (blue) serial contraction signals. Negative trends in work and associated power functions show that serial strategies underwent refrigeration cycles of increasingly greater magnitude to maintain constant entropy levels. **(C,D)** Trends in mean average efficiencies and coefficients of performance for modal (gold and pink, respectively) and non-modal (purple and brown, respectively) serial contraction signals corresponded with work and power functions, confirming ciliates behave like Szilárd engines employing refrigeration cycles when necessary to correct signaling strategy errors. **(E)** Higher trends in the mean average fidelity of Boltzmann entropy between strategy planning and execution stages indicate repetition coding was more successful in protecting modal (red) than non-modal (green) serial contraction strategies from error. Ciliates learn via Markov Szilárd-engine processes to improve fidelity between strategy planning and execution stages for all strategies for each corrective. All trend lines for panels **(B)** through **(E)** are fitted to second-order polynomial equations. Black trend lines represent composites of modal and non-modal serial strategy trends. Symbol T in panels **(A)** and **(B)** is temperature in °K.

Higher fidelity for initial low contraction responders, as compared to other levels of behavioral responsiveness, means fewer signal errors in magnitude and/or frequency occurred in specific serial strategies for this group of protozoa. Some amount of error corrupted strategy planning and execution for all initial responsiveness groups, as perfect mean average fidelities of 1 were not attained by low, medium, and high contraction responders (see above subsection). However, the effectiveness of microbes to employ repetition coding, which should limit the magnitude and frequency of errors incurred during signal production, might account for observed group disparities in signal-production fidelity by differentially affecting individual strategies. Preceding analyses were not sensitive to such possibilities since planned comparisons were conducted across modal and non-modal strategies. But a weighted 3 × 8 repeated-measures factorial analysis of difference scores, determined from mean average planning-stage Boltzmann entropies introduced with estimated single bit-flip errors and corresponding unaltered observed mean average execution-stage Boltzmann entropies, showed that the effectiveness of repetition coding to safeguarded all eight serial contraction strategies processed over time significantly differed according to responsiveness group [*F*_(2, 1368)_ = 22.23, *p* = 3.16 × 10^−10^] and mating strategy type [*F*_(7, 1368)_ = 17.97, *p* = 5.79 × 10^−23^] with a significant interaction effect [*F*_(14, 1368)_ = 2.82, *p* = 3.50 × 10^−4^]. Partial comparisons yielded significant respective simple effects for responsiveness group, strategy type, and factor interactions for initial low and medium contraction responders [*F*_(1, 912)_ = 17.66, *p* = 2.90 × 10^−5^, $\bar{x}$_*L*_ = 3.59 × 10^−25^ SEM = 4.41 × 10^−49^, $\bar{x}$_*M*_ = 1.83 × 10^−25^ SEM = 4.81 × 10^−49^; *F*_(7, 912)_ = 18.69, *p* = 2.24 × 10^−23^; *F*_(7, 912)_ = 1.83, *p* = 0.079], initial medium and high contraction responders [*F*_(1, 912)_ = 5.34, *p* = 0.021, $\bar{x}$_*M*_ = 1.83 × 10^−25^ SEM = 4.81 × 10^−49^, $\bar{x}$_*H*_ = 8.62 × 10^−26^ SEM = 3.96 × 10^−49^; *F*_(7, 912)_ = 8.27, *p* = 8.38 × 10^−10^; *F*_(7, 912)_ = 3.48, *p* = 1.10 × 10^−3^], and initial low and high contraction responders [*F*_(1, 912)_ = 45.16, *p* = 3.20 × 10^−11^, $\bar{x}$_*L*_ = 3.59 × 10^−25^ SEM = 4.41 × 10^−49^, $\bar{x}$_*H*_ = 8.62 × 10^−26^ SEM = 3.96 × 10^−49^; *F*_(7, 912)_ = 11.80, *p* = 1.96 × 10^−14^; *F*_(7, 912)_ = 3.17, *p* = 2.53 × 10^−3^]. Initial low contraction responders employed repetition coding with better success than other initial contraction responsiveness groups, as denoted by the higher mean average difference score per strategy per learning trial (e.g., $\bar{x}$*_*L*_* = 3.59 × 10^−25^ SEM = 4.41 × 10^−49^). In addition, if repetition coding had failed to protect signals between strategy planning and execution stages, then the Boltzmann entropies of executed strategies would have significantly exceeded estimated error-threshold values. Difference scores proved this was not the case. Superior use of repetition coding also should be revealed in the number of executed strategies which subtended bit-flip error thresholds. Two-tailed paired comparisons showed that initial low contraction responders were again better in this computational respect, with the absolute mean average Boltzmann entropies for 6 of 8 executed contraction signals falling significantly below single bit-flip error thresholds (Figure 2A and Table 1). Whereas, absolute mean average Boltzmann entropies for half of the executed serial mating signals executed by initial medium contraction responders fell below single bit-flip error thresholds (Figure 3A and Table 1). Absolute mean average Boltzmann entropies for only 3 of 8 serial replies executed by initial high contraction responders fell below single bit-flip error thresholds (Figure 4A and Table 1).

**Table 1:** **Comparisons of contraction Boltzmann entropies to bit-flip error thresholds**.

**Strategy**	**Parametric statistic**	***p*-value**	$\bar{x}$_*f*_ ± SEM^*^	**$\bar{x}$_*E*_ ± SEM^†^**
**LOW CONTRACTION RESPONDERS**				
(0,0,0)	*t*_(55)_ = −16.858	2.18 × 10^−23^	8.93 × 10^−25^ ± 2.06 × 10^−50^	3.05 × 10^−25^ ± 4.00 × 10^−50^
(0,0,1)	*t*_(51)_ = −5.778	4.56 × 10^−7^	1.15 × 10^−24^ ± 1.48 × 10^−49^	4.59 × 10^−25^ ± 4.76 × 10^−49^
(0,1,0)	*t*_(50)_ = −4.501	4.06 × 10^−5^	1.20 × 10^−24^ ± 1.40 × 10^−49^	6.7 × 10^−25^ ± 4.76 × 10^−49^
(1,0,0)	*t*_(51)_ = −7.561	7.11 × 10^−10^	1.24 × 10^−25^ ± 1.42 × 10^−49^	4.07 × 10^−25^ ± 4.73 × 10^−49^
(0,1,1)	*t*_(30)_ = −1.165	0.25	1.08 × 10^−24^ ± 1.60 × 10^−49^	8.93 × 10^−25^ ± 7.75 × 10^−49^
(1,1,0)	*t*_(29)_ = −5.933	1.92 × 10^−6^	1.20 × 10^−24^ ± 1.10 × 10^−49^	6.67 × 10^−25^ ± 1.820 × 10^−49^
(1,0,1)	*t*_(28)_ = −2.414	0.02	1.10 × 10^−24^ ± 2.24 × 10^−49^	8.63 × 10^−25^ ± 3.59 × 10^−49^
(1,1,1)	*t*_(14)_ = 0.500	0.63	7.86 × 10^−25^ ± 5.34 × 10^−50^	8.90 × 10^−25^ ± 6.12 × 10^−50^
**MEDIUM CONTRACTION RESPONDERS**				
(0,0,0)	*t*_(53)_ = −15.588	1.92 × 10^−21^	9.61 × 10^−25^ ± 3.25 × 10^−50^	2.64 × 10^−25^ ± 3.26 × 10^−50^
(0,0,1)	*t*_(56)_ = −2.256	0.028	7.97 × 10^−25^ ± 7.55 × 10^−50^	5.71 × 10^−25^ ± 4.02 × 10^−49^
(0,1,0)	*t*_(54)_ = −1.788	0.079	8.77 × 10^−25^ ± 1.60 × 10^−49^	6.86 × 10^−25^ ± 4.65 × 10^−49^
(1,0,0)	*t*_(52)_ = −4.112	1.40 × 10^−4^	9.65 × 10^−25^ ± 1.47 × 10^−49^	5.29 × 10^−25^ ± 4.77 × 10^−49^
(0,1,1)	*t*_(47)_ = −0.123	0.902	6.90 × 10^−25^ ± 1.35 × 10^−49^	6.76 × 10^−25^ ± 6.69 × 10^−49^
(1,1,0)	*t*_(47)_ = −1.367	0.178	8.43 × 10^−25^ ± 2.46 × 10^−49^	6.82 × 10^−25^ ± 7.35 × 10^−49^
(1,0,1)	*t*_(46)_ = −0.699	0.488	6.72 × 10^−25^ ± 1.54 × 10^−49^	7.31 × 10^−25^ ± 1.68 × 10^−49^
(1,1,1)	*t*_(37)_ = 2.514	0.016	6.39 × 10^−25^ ± 6.49 × 10^−50^	9.10 × 10^−25^ ± 4.75 × 10^−49^
**HIGH CONTRACTION RESPONDERS**				
(0,0,0)	*t*_(38)_ = −0.622	0.538	7.72 × 10^−25^ ± 1.22 × 10^−49^	6.95 × 10^−25^ ± 4.19 × 10^−49^
(0,0,1)	*t*_(40)_ = −1.635	0.11	7.30 × 10^−25^ ± 1.89 × 10^−49^	4.97 × 10^−25^ ± 6.18 × 10^−49^
(0,1,0)	*t*_(41)_ = −1.063	0.294	8.31 × 10^−25^ ± 2.65 × 10^−49^	6.85 × 10^−25^ ± 5.44 × 10^−49^
(1,0,0)	*t*_(43)_ = −2.505	0.016	8.40 × 10^−25^ ± 2.97 × 10^−49^	4.76 × 10^−25^ ± 5.65 × 10^−49^
(0,1,1)	*t*_(38)_ = −2.276	0.029	6.61 × 10^−25^ ± 1.75 × 10^−49^	4.86 × 10^−25^ ± 1.75 × 10^−49^
(1,1,0)	*t*_(39)_ = −3.889	3.801 × 10^−4^	6.77 × 10^−25^ ± 1.73 × 10^−49^	4.00 × 10^−25^ ± 1.91 × 10^−49^
(1,0,1)	*t*_(41)_ = −1.966	0.060	6.65 × 10^−25^ ± 1.87 × 10^−49^	7.95 × 10^−25^ ± 2.68 × 10^−49^
(1,1,1)	*t*_(43)_ = 1.684	0.10	5.47 × 10^−25^ ± 7.64 × 10^−50^	7.12 × 10^−25^ ± 3.52 × 10^−49^

* f subscript denotes bit-flip error;

† E subscript denotes executed.

### Ciliate preparedness determined error-syndrome diagnosis and correction of modal contraction mating signals

Since initial low, medium, and high contraction responders utilized repetition coding with varying success and because that success differed for individual strategies, different groups of initial contraction responsiveness might be better at detecting and correcting error syndromes for specific serial strategies. Earlier analyses were not sensitive to such possibilities as planned comparisons were collapsed across modal and non-modal strategies. Szilárd engine characteristics were consequently evaluated for the separate processing of modal and non-modal serial contraction strategies signaling mating fitness. Single-factor analyses between groups confirmed mean average work [*F*_(2, 171)_ = 16.10, *p* = 3.94 × 10^−7^; $\bar{x}$_*L*_ = 5.03 × 10^−26^ SEM = 5.39 × 10^−49^, $\bar{x}$_*M*_ = −1.30 × 10^−25^ SEM = 8.42 × 10^−50^, $\bar{x}$_*H*_ = −4.80 × 10^−25^ SEM = 6.45 × 10^−49^], efficiency [*F*_(2, 171)_ = 20.74, *p* = 8.60 × 10^−9^; $\bar{x}$_*L*_ = 6.92 × 10^−3^ SEM = 1.23 × 10^−2^, $\bar{x}$_*M*_ = −4.15 × 10^−2^ SEM = 4.10 × 10^−3^, $\bar{x}$_*H*_ = −1.51 × 10^−1^ SEM = 3.8 × 10^−1^], and fidelity [*F*_(2, 171)_ = 77.80, *p* = 9.37 × 10^−25^; $\bar{x}$_*L*_ = 0.492 SEM = 0.018, $\bar{x}$_*M*_ = 0.322 SEM = 0.019, $\bar{x}$_*H*_ = 0.218 SEM = 0.006] for modal strategies significantly differed for initial behavioral responsiveness. The lower mean average work and higher mean average efficiency and fidelity of low contraction responders, as compared to other responsiveness groups, indicates these ciliates were capable of limiting the occurrence of errors in modal signals between strategy planning and execution stages and, therefore, required less work for error-syndrome correction. Ciliates with initial high contraction responsiveness, however, had lower mean average fidelity and needed higher negative mean average work and efficiency to correct errors in modal signals with refrigeration cycles. In contrast to modal strategy production, Szilárd-engine operational characteristics did not differ for non-modal strategies processed by ciliates of varying initial responsiveness. Two-tailed paired comparisons within levels of behavioral responsiveness further revealed that only mean average efficiency and fidelity tended to be significantly higher for modal, as opposed to non-modal, mating signals processed by ciliates of initial low [ε: *t*_(57)_ = 2.883, *p* = 5.54 × 10^−3^, $\bar{x}$_*M*_ = −0.007 SEM = 0.012, $\bar{x}$_*n*_ = −0.085 SEM = 0.047; *F*: *t*_(57)_ = 28.093, *p* = 4.43 × 10^−35^, $\bar{x}$_*M*_ = 0.492 SEM = 0.019, $\bar{x}$_*n*_ = 0.149 SEM = 0.005], medium [ε : *t*_(57)_ = 3.154, *p* = 2.57 × 10^−3^, $\bar{x}$_*M*_ = −0.042 SEM = 0.004, $\bar{x}$_*n*_ = −0.117 SEM = 0.033; *F*: *t*_(57)_ = 14.326, *p* = 1.52 × 10^−20^, $\bar{x}$_*M*_ = 0.322 SEM = 0.019, $\bar{x}$_*n*_ = 0.148 SEM = 0.006], and high [ε : *t*_(57)_ = −3.846, *p* = 3.06 × 10^−4^, $\bar{x}$_*M*_ = −0.151 SEM = 0.038, $\bar{x}$_*n*_ = −0.042 SEM = 0.014; *F*: *t*_(57)_ = 5.324, *p* = 1.79 × 10^−6^, $\bar{x}$_*M*_ = 0.218 SEM = 0.006, $\bar{x}$_*n*_ = 0.163 SEM = 0.012] contraction responsiveness.

### Ciliates learned to change Szilárd engine performance when processing contraction mating signals

Mean average work, power, efficiency, coefficient of performance, and fidelity measures tended to vary over time for the processing of both modal and non-modal signals by all initial behavioral responsiveness groups. However, when comparing each of these Szilárd engine performance indices against its corresponding single-frame-advanced series of scores, single-factor analysis showed that only modal strategy fidelity improved significantly with time for ciliates of low [Figure 2; *F*_(56, 57)_ = 7.124, *p* = 3.05 × 10^−12^], medium [Figure 3; *F*_(56, 57)_ = 10.744, *p* = 2.60 × 10^−16^], and high [Figure 4; *F*_(56, 57)_ = 2.567, *p* = 2.59 × 10^−4^] contraction responsiveness. These findings demonstrate that protozoa learned to change the fidelity of modal signals via a Markov process over iterative engine cycles. Small negative trends in the refinement of engine work and efficiency between learning trials likely account for this result and function as refrigeration cycles. Similar effects were found for the iterative processing of non-modal serial contraction strategies. Single-factor analysis established that respective non-modal strategy fidelity and efficiency significantly changed with time for low [Figure 2; *F*_(56, 57)_ = 6.663, *p* = 1.29 × 10^−11^; *F*_(56, 57)_ = 2.648, *p* = 1.71 × 10^−4^], medium [Figure 3; *F*_(56, 57)_ = 4.985, *p* = 4.70 × 10^−9^; *F*_(56, 57)_ = 1.912, *p* = 8.01 × 10^−3^], and high [Figure 4; *F*_(56, 57)_ = 12.955, *p* = 2.82 × 10^−18^; *F*_(56, 57)_ = 3.038, *p* = 2.40 × 10^−5^] contraction responders. In addition, the mean average value of work for ciliates of initial high responsiveness significantly changed with time [*F*_(56, 57)_ = 1.978, *p* = 5.68 × 10^−3^], suggesting both learned negative changes in engine efficiency and work improved fidelity between non-modal strategy planning and execution stages with each corrective refrigeration cycle.

### Ciliate preparedness determined success of repetition codes to noise-protect reversal mating signals

**Figure 5:**
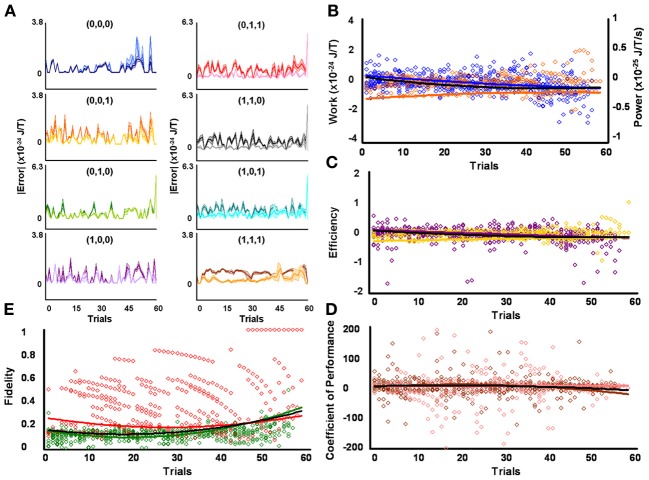
**Initial medium responders learn to diagnose and correct classical errors in executed reversal strategies signaling mating fitness. (A)** Repetition coding prevented errors in executed serial reversal signals from exceeding estimated single bit-flip errors in absolute mean average Boltzmann entropy [executed (light blue) and bit-flipped (dark blue) strategy (0,0,0), executed (light orange) and bit-flipped (dark orange) strategy (0,0,1), executed (lime green) and bit-flipped (green) strategy (0,1,0), executed (lavender) and bit-flipped (purple) strategy (1,0,0), executed (pink) and bit-flipped (red) strategy (0,1,1), executed (gray) and bit-flipped (black) strategy (1,1,0), executed (turquoise) and bit-flipped (teal) strategy (1,0,1), executed (gold) and bit-flipped (brown) strategy (1,1,1)]. **(B)** Differences in mean average Boltzmann entropy between strategy planning and execution stages were expelled by ciliates when learning to detect and correct errors in modal (orange) and non-modal (blue) serial reversal signals. Negative trends in work and associated power functions show that serial strategies underwent refrigeration cycles of increasingly greater magnitude to maintain constant entropy levels. **(C,D)** Trends in mean average efficiencies and coefficients of performance for modal (gold and pink, respectively) and non-modal (purple and brown, respectively) serial reversal signals corresponded with work and power functions, confirming ciliates behave like Szilárd engines employing refrigeration cycles when necessary to correct signaling strategy errors. **(E)** Higher trends in the mean average fidelity of Boltzmann entropy between strategy planning and execution stages indicate repetition coding was more successful in protecting modal (red) than non-modal (green) serial reversal strategies from error. Ciliates learn via Markov Szilárd-engine processes to improve fidelity between strategy planning and execution stages for all strategies for each corrective. All trend lines for panels **(B)** through **(E)** are fitted to second-order polynomial equations. Black trend lines represent composites of modal and non-modal serial strategy trends. Symbol T in panels **(A)** and **(B)** is temperature in °K.

**Figure 6:**
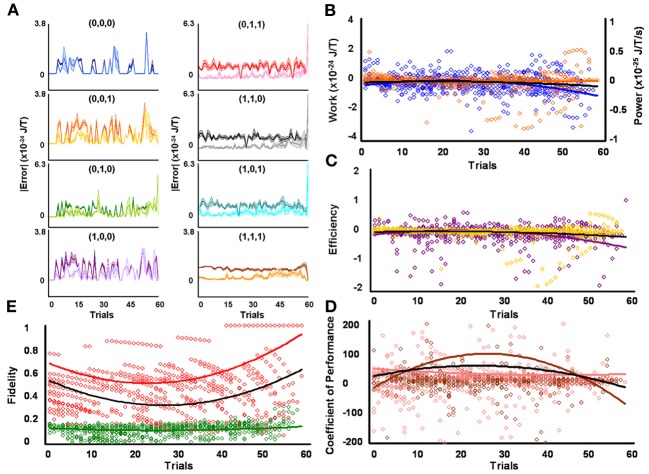
**Initial high responders learn to diagnose and correct classical errors in executed reversal strategies signaling mating fitness. (A)** Repetition coding prevented errors in executed serial reversal signals from exceeding estimated single bit-flip errors in absolute mean average Boltzmann entropy [executed (light blue) and bit-flipped (dark blue) strategy (0,0,0), executed (light orange) and bit-flipped (dark orange) strategy (0,0,1), executed (lime green) and bit-flipped (green) strategy (0,1,0), executed (lavender) and bit-flipped (purple) strategy (1,0,0), executed (pink) and bit-flipped (red) strategy (0,1,1), executed (gray) and bit-flipped (black) strategy (1,1,0), executed (turquoise) and bit-flipped (teal) strategy (1,0,1), executed (gold) and bit-flipped (brown) strategy (1,1,1)]. **(B)** Differences in mean average Boltzmann entropy between strategy planning and execution stages were expelled by ciliates when learning to detect and correct errors in modal (orange) and non-modal (blue) serial reversal signals. Negative trends in work and associated power functions show that serial strategies underwent refrigeration cycles of increasingly greater magnitude to maintain constant entropy levels. **(C,D)** Trends in mean average efficiencies and coefficients of performance for modal (gold and pink, respectively) and non-modal (purple and brown, respectively) serial reversal signals corresponded with work and power functions, confirming ciliates behave like Szilárd engines employing refrigeration cycles when necessary to correct signaling strategy errors. **(E)** Higher trends in the mean average fidelity of Boltzmann entropy between strategy planning and execution stages indicate repetition coding was more successful in protecting modal (red) than non-modal (green) serial reversal strategies from error. Ciliates learn via Markov Szilárd-engine processes to improve fidelity between strategy planning and execution stages for all strategies for each corrective. All trend lines for panels **(B)** through **(E)** are fitted to second-order polynomial equations. Black trend lines represent composites of modal and non-modal serial strategy trends. Symbol T in panels **(A)** and **(B)** is temperature in °K.

As with contraction responders, some amount of error corrupted strategy planning and execution for all initial reversal responsiveness groups. Initial high reversal responders, however, produced higher mean average fidelity between strategy planning and execution stages when compared to ciliates grouped by initial medium reversal responsiveness. This finding again suggests specific serial strategies processed by these microbes incurred lower error magnitudes and/or frequencies. The effectiveness of protozoa to employ repetition coding, which should limit the magnitude and frequency of errors arising during signal production, probably caused some of the observed group variations in fidelity measures by differentially affecting individual strategies. Indeed, weighted 2 × 8 repeated-measures factorial analysis of difference scores, calculated from mean average planning-stage Boltzmann entropies introduced with estimated single bit-flip errors and corresponding unaltered observed mean average execution-stage Boltzmann entropies, demonstrated that effectiveness of repetition coding to safeguarded all eight serial reversal strategies significantly differed according to responsiveness group [*F*_(1, 912)_ = 32.40, *p* = 1.69 × 10^−8^; $\bar{x}$_*M*_ = 2.69 × 10^−25^ SEM = 4.15 × 10^−49^, $\bar{x}$_*H*_ = 5.04 × 10^−25^ SEM = 5.12 × 10^−49^] and mating strategy type [*F*_(7, 912)_ = 22.61, *p* = 2.37 × 10^−28^]. Initial high reversal responders employed repetition coding with better success than initial medium reversal responders, as evidenced by the higher mean average difference score per strategy per learning trial (i.e., $\bar{x}$_*H*_ = 5.04 × 10^−25^ SEM = 5.12 × 10^−49^). Difference scores verified that mean average Boltzmann entropies of executed reversal signals failed to exceed estimated error threshold values and, therefore that repetition coding protected signals from noise during signal production. Superior use of repetition coding also should be revealed in the number of executed strategies which subtended bit-flip error thresholds. But two-tailed paired comparisons showed that initial medium and high reversal responders were equally good in this computational respect, with the absolute mean average Boltzmann entropies for 6 of 8 mating signals executed by medium (Figure 5A and Table 2) and high (Figure 6A and Table 2) responsiveness groups falling significantly below single bit-flip error thresholds.

**Table 2:** **Comparisons of reversal Boltzmann entropies to bit-flip error thresholds**.

**Strategy**	**Parametric statistic**	***p*-value**	$\bar{x}$_*f*_ ± SEM^*^	**$\bar{x}$_*E*_ ± SEM^†^**
**MEDIUM REVERSAL RESPONDERS**				
(0,0,0)	*t*_(34)_ = 1.485	0.147	4.77 × 10^−25^ ± 8.59 × 10^−50^	6.08 × 10^−25^ ± 3.93 × 10^−49^
(0,0,1)	*t*_(44)_ = −4.443	5.93 × 10^−5^	7.77 × 10^−25^ ± 2.55 × 10^−49^	4.68 × 10^−25^ ± 9.75 × 10^−50^
(0,1,0)	*t*_(43)_ = 0.20	0.842	7.20 × 10^−25^ ± 2.02 × 10^−49^	7.44 × 10^−25^ ± 5.98 × 10^−49^
(1,0,0)	*t*_(38)_ = −3.320	1.997 × 10^−3^	6.74 × 10^−25^ ± 1.99 × 10^−49^	4.30 × 10^−25^ ± 9.18 × 10^−50^
(0,1,1)	*t*_(50)_ = −6.039	1.910 × 10^−7^	1.06 × 10^−24^ ± 1.75 × 10^−49^	4.24 × 10^−25^ ± 4.88 × 10^−49^
(1,1,0)	*t*_(51)_ = −4.652	2.36 × 10^−5^	9.74 × 10^−25^ ± 1.47 × 10^−49^	4.31 × 10^−25^ ± 4.71 × 10^−49^
(1,0,1)	*t*_(51)_ = −3.270	1.928 × 10^−3^	1.03 × 10^−24^ ± 1.31 × 10^−49^	6.39 × 10^−25^ ± 4.51 × 10^−49^
(1,1,1)	*t*_(55)_ = −10.843	2.84 × 10^−15^	8.06 × 10^−25^ ± 4.18 × 10^−50^	3.38 × 10^−25^ ± 5.64 × 10^−50^
**HIGH REVERSAL RESPONDERS**				
(0,0,0)	*t*_(26)_ = −0.580	0.567	8.19 × 10^−25^ ± 1.13 × 10^−49^	7.55 × 10^−25^ ± 4.68 × 10^−49^
(0,0,1)	*t*_(39)_ = −6.552	8.89 × 10^−8^	1.13 × 10^−24^ ± 3.12 × 10^−49^	5.91 × 10^−25^ ± 2.01 × 10^−49^
(0,1,0)	*t*_(41)_ = −1.843	0.073	1.01 × 10^−24^ ± 2.22 × 10^−49^	7.29 × 10^−25^ ± 6.76 × 10^−49^
(1,0,0)	*t*_(41)_ = −4.256	1.18 × 10^−4^	1.03 × 10^−24^ ± 2.62 × 10^−49^	6.18 × 10^−25^ ± 1.95 × 10^−49^
(0,1,1)	*t*_(55)_ = −9.680	1.77 × 10^−13^	1.44 × 10^−25^ ± 1.01 × 10^−49^	4.79 × 10^−25^ ± 4.43 × 10^−49^
(1,1,0)	*t*_(57)_ = −8.096	4.76 × 10^−11^	1.31 × 10^−24^ ± 9.44 × 10^−50^	4.34 × 10^−25^ ± 4.49 × 10^−49^
(1,0,1)	*t*_(56)_ = −6.860	5.90 × 10^−9^	1.31 × 10^−24^ ± 9.64 × 10^−50^	5.68 × 10^−25^ ± 4.29 × 10^−49^
(1,1,1)	*t*_(57)_ = −17.746	6.94 × 10^−25^	8.82 × 10^−25^ ± 2.75 × 10^−50^	2.82 × 10^−25^ ± 3.39 × 10^−50^

* f subscript denotes bit-flip error;

† E subscript denotes executed.

### Ciliate preparedness determined error-syndrome diagnosis and correction of modal reversal mating signals

Initial medium and high reversal responders employed repetition coding with varying success for different individual strategies. Therefore, different groups of initial reversal responsiveness might be better at detecting and correcting error syndromes for specific serial strategies. As with contraction responders, Szilárd engine characteristics were consequently evaluated for the separate processing of modal and non-modal serial reversal strategies signaling mating commitment. Single-factor analyses between groups confirmed only mean average fidelity [*F*_(1, 114)_ = 20.17, *p* = 1.71 × 10^−5^; $\bar{x}$_*M*_ = 0.408 SEM = 0.018, $\bar{x}$_*H*_ = 0.509 SEM = 0.011] for modal strategies significantly differed for initial behavioral responsiveness. These results contrast those observed for contraction responders, where modal strategy work, efficiency, and fidelity differed for level of behavioral responsiveness. In addition, Szilárd engine operational characteristics did not differ for non-modal reversal strategies processed by ciliates of varying initial responsiveness, a finding consistent with that for contraction responders. Two-tailed paired comparisons within levels of behavioral responsiveness further revealed that mean average efficiency and fidelity tended to be significantly elevated for modal, as opposed to non-modal, mating signals processed by ciliates of initial medium [*ε* : *t*_(57)_ = 2.113, *p* = 0.039, $\bar{x}$_*M*_ = −0.013 SEM = 0.074, $\bar{x}$_*n*_ = −0.053 SEM = 0.030; *F*: *t*_(57)_ = 18.169, *p* = 2.21 × 10^−25^, $\bar{x}$_*M*_ = 0.408 SEM = 0.018, $\bar{x}$_*n*_ = 0.151 SEM = 0.007] and high [*ε* : *t*_(57)_ = 3.125, *p* = 2.80 × 10^−3^, $\bar{x}$_*M*_ = −0.035 SEM = 0.026, $\bar{x}$_*n*_ = −0.110 SEM = 0.044; *F*: *t*_(57)_ = 47.262, *p* = 2.05 × 10^−47^, $\bar{x}$_*M*_ = 0.508 SEM = 0.011, $\bar{x}$_*n*_ = 0.144 SEM = 0.006] reversal responsiveness.

### Ciliates learned to change Szilárd engine performance when processing reversal mating signals

Mean average work, power, efficiency, coefficient of performance, and fidelity measures tended to vary over time for the processing of both modal and non-modal signals by all initial reversal responsiveness groups. However, when comparing each of these Szilárd engine performance indices against its corresponding single-frame-advanced series of scores, single-factor analysis showed that modal strategy fidelity improved significantly with time for ciliates of medium [Figure 5; *F*_(56, 57)_ = 5.39, *p* = 1.02 × 10^−9^] and high [Figure 6; *F*_(56, 57)_ = 11.45, *p* = 5.71 × 10^−17^] reversal responsiveness and that modal strategy work and efficiency changed with time for high reversal responders [Figure 6; *W*: *F*_(56, 57)_ = 3.14, *p* = 1.43 × 10^−5^; ε: *F*_(56, 57)_ = 1.58, *p* = 0.045]. These findings demonstrate that protozoa learned to change the fidelity of modal signals via a Markov process over iterative engine cycles. Small negative trends in the refinement of engine work and efficiency between learning trials likely account for this result and function as refrigeration cycles. Similar effects were found for the iterative processing of non-modal serial contraction strategies. Single-factor analysis established that respective non-modal strategy fidelity and efficiency significantly changed with time for initial medium [Figure 5; *F*: *F*_(56, 57)_ = 5.04, *p* = 3.75 × 10^−9^; ε : *F*_(56, 57)_ = 1.83, *p* = 0.013] and high [Figure 6; *F*: *F*_(56, 57)_ = 8.75, *p* = 3.11 × 10^−14^; ε : *F*_(56, 57)_ = 2.087, *p* = 3.21 × 10^−3^] reversal responders. In addition, the mean average value of work for ciliates of initial medium responsiveness significantly changed with time [*F*_(56, 57)_ = 1.69, *p* = 0.025], suggesting both learned negative changes in engine efficiency and work improved fidelity between non-modal strategy planning and execution stages with each corrective refrigeration cycle.

## Conclusions

### Possible evolutionary significance of classical error diagnosis and correction

Many of the survival and reproductive achievements of colonial bacteria, algae, fungi, and protozoa, like other kinds of organisms, are dependent upon genetically predisposed, epigenetically modifiable, and traditionally learned social skills that enhance an individual microbe's ability to communicate and interact with others. Good social skills often make groups of microbes more adaptable through coordinated hunting and foraging, mate selection, altruistic suicide, assisted reproduction, induced defenses, and additional behaviors (e.g., Ricci, 1990; Crespi, 2001; Ben-Jacob et al., 2004; Clark, 2010a, 2012a, 2013). But without proper methods to control errors in social interactions, the ecological success of social species, subspecies, collectives, kin, and individuals would deteriorate to levels indistinct from those of solitary organisms. Although computational fault-tolerance figures to substantially impact a number of microbial behaviors, such as intra- and intermate selection (Clark, 2011a, 2012a), which contribute to transmission and virulence of infectious diseases and major evolutionary transitions, scientists have made little progress in elucidating this crucial aspect of information processing. Employing an information theoretic approach and simulated mating contexts, it has been shown here for the first time that ciliates learn to behave as Szilárd engines to diagnose and correct classical information errors incurred throughout the production of serial behavioral strategies useful for signaling mating availability and prowess during perimating displays. Regardless of initial behavioral responsiveness, and therefore how a ciliate perceives and reacts to its environment, classical three-bit repetition codes protected processed mating signals from exceeding single bit-flip error thresholds. Ciliate preparedness, however, did determine the effectiveness of Szilárd engine cycles to both detect deviations in Boltzmann entropy and recover corrupted information as measured by signal fidelity between strategy planning and execution stages. Initial low contraction and high reversal responders tended to be better than other ciliates at maintaining overall elevated magnitudes of fidelity and at learning to improve fidelity of modal strategies across learning trials.

Modal strategies, particularly those of ideal conspicuous consumption and prudent savings, convey highly significant ecological information about mating competency, such as respective harder-to-get and easier-to-get assurances (Clark, 2010a, 2012a, 2013). Initial low contraction and high reversal responders signal modal strategies with greater frequency, briefly learning to only switch to less preferred alternative non-modal strategies when trying to persuade suitors and cheat rivals (Clark, 2010a). Corrupted modal behavioral strategies thus represent serious disadvantages specific to ciliates prepared to advertise extreme levels of mating fitness and readiness prior to paired reproduction. The reproductive value of modal strategies, in contrast to non-modal strategies, for these ciliates and, to a lesser degree, ciliates of different behavioral responsiveness either conferred or coincided with more efficient Szilárd engine cycles, limiting the amount of noise that occurred during signal production and, consequently, the magnitude and frequency of errors which plagued executed mating replies. Preventing error syndromes with refinements in heat-engine efficiency is a clearly different process than learning to increase or decrease levels of engine work and power when diagnosing and correcting errors. Ciliates may lower the probability of generating errors during signal production with improved heat-engine efficiency or they may reject errors during signal production by increasing negative work and coefficient of performance obtained from refrigerator cycles. For instance, improved mating signal fidelity between non-modal behavioral strategy planning and execution stages resulted from iterative Hebbian-like changes in both engine efficiency and work. Non-modal serial strategies are comparably more susceptible to information degradation because they are less resistant to bit-flip errors. For these sorts of courting replies, protozoa mainly exerted extra effort to diagnose and correct incurred errors with refrigeration cycles rather than simply adjusting the power-stoke efficiency of heat engine cycles.

Moreover, although the present studies were interpreted in terms of mating contexts, similar error-syndrome diagnosis and correction procedures integrating repetition coding with Szilárd engine performance likely safeguard the information content and specificity of other forms of ciliate behavioral communications and might be universally used by all microbes during social situations. The particular experimental paradigm employed for this study exploited the mechanosensation of *S. ambiguum* and the importance of mechanosensation for heterotrich mating interactions. Mechanosensation, while mediated by disparate transduction mechanisms (cf. Martinac et al., 2008), is a phylogenetically conserved sensory modality of single-celled organisms. Bacteria, algae, fungi, and protozoa other than heterotrich ciliates also rely on mechanosensation for sensing osmotic stress, prey or predators, reproductive interactions, foraging, cell aggregation, surface contours, and a variety of other cell physiologic and ecological conditions. Chemical communications serve as a primary means for microbes to exchange social information (Crespi, 2001; Hirt et al., 2002; Winans and Bassler, 2002; Ben-Jacob et al., 2004; Wolf et al., 2008; Koseska et al., 2009; Lehner, 2010; Marijuán et al., 2010), but high-fidelity mechanical communication is nonetheless critical for coordinating collective behaviors and the spatial organization of colonies. Accordingly, use of classical repetition codes by microbes to protect serial mechanical messages and replies from ambient noise and to diagnose and correct errors in such signals before they are transmitted represents a possible widespread evolutionary adaptation in the computational abilities of bacteria to protozoa. Before firm conclusions can be drawn with respect to the universality of error-correction codes, microbes from different and diverse taxa need to be evaluated. Nevertheless, repetition coding of sensitive social information is a commonly evolved strategy used in the Animal Kingdom to simply secure or encrypt publicly conveyed signals from eavesdroppers (cf. Hauser, 1997). The repetitive vocalizations and behavioral displays of primates and birds perhaps best exemplify this evolutionary trait, but examples can be found for many vertebrate and invertebrate taxa. It is not unreasonable to predict that many different taxa of microbes also optimize their social fitness through evolved classical repetition codes.

### Possible biological mechanisms of classical error diagnosis and correction

The set of studies reported here concentrated on assessing the phenomenological computational attributes of classical error diagnosis and correction of protozoa. Future research will have to address the biological mechanisms underlying such phenomena. One possible mechanism involves learned modification of intracellular Ca^2+^ entry, homeostasis, and feedback regulation, which can account for previously observed classical and quantum computational phases (Clark, 2010a,b,c,d,e, 2011a,b,c, 2012a,b). In ciliates, as well as bacteria, algae, and fungi, free intracellular Ca^2+^ acts as a response regulator critical for sensing and reacting to environmental stimuli (cf. Baneutt, 1998; Dominguez, 2004; Martinac et al., 2008; Clark, 2010a), such as preconjugal cell-cell touches. Mechanical pressures experienced by ciliates during social strategizing stimulate Ca^2+^ entry into a cell via cooperative G-protein-coupled mechanoreceptors and voltage-gated channels. Rising Ca^2+^ concentrations autocatalytically trigger further Ca^2+^ release from intracellular stores that form compartmentalized networks throughout the cortical and subadjacent endoplasmic regions containing cytoskeletal myonemes and axonemes required to elicit cell motility and social interactions. These fire-diffuse-fire reactions are believed to underlie observed increases in ciliate strategy searches following strategy planning (Clark, 2010a,b,c,d,e, 2011b,c) and possibly function as digital information channels (Plieth, 2005). As the reaction kinetics of Ca^2+^ waves become restricted by the duration of Ca^2+^ mobilization, wave velocity quickens in quadratic proportion to the classical Ca^2+^ diffusion coefficient (Ponce-Dawson et al., 1999), matching the improved efficiency of Grover's quantum search algorithm over classical processes without evoking use of quantum diffusion terms (Clark, 2011c, 2012b). Furthermore, distinctive spatiotemporal patterns of activated intracellular Ca^2+^ stores crosstalk with other response regulator systems, such as pheromone transduction pathways (Miyake, 1981; Nielsen and Heitman, 2007) and cAMP pathways (Siso-Nadal et al., 2009), to likely integrate and “bind” salient information into coherent chemical states or associative memory circuits. Such internal representations are probably selectively activated and modified by further external and internal stimulation and the subsequent differential spread and speed of fire-diffuse-fire Ca^2+^ waves caused by variable Ca^2+^ buffering and uptake, periods of Ca^2+^ mobilization, distances between calcium storage sites and effector systems, and phosphatase- and protein-kinase-dependent feedback control over Ca^2+^-conducting membrane receptors (Chen et al., 2008, 2009; De Pitta et al., 2008, 2009; Clark, 2010a,b,c,d,e, 2011a,b,c, 2012a,b).

In addition, limited evidence suggests the digital representation of processed mating signals, from planning to execution stages, can be encoded, intracellularly transmitted, and stored by free intracellular Ca^2+^ (Plieth, 2005), calcium-calmodulin kinase II (CaMKII) holoenzyme (Hameroff et al., 2010), and other Ca^2+^-related substrate broadly distributed across affector-effector systems. Individual molecules conveying bitwise information may form higher-order bytes at large concentrations and/or with molecular complexes, such as that reported for six-domain CaMKII encoding of microtubule lattices (Hameroff et al., 2010). However, the molecular storage and other operational properties of cyctoskeleton in ciliates figure to be different than that of higher eukaryotes, where, for example, microtubules affect the geometry of dendritic spines and synaptic cleft width (Dent et al., 2011), the intracellular transport of ions and molecules (Craddock et al., 2010; Priel et al., 2010), and, therefore, the efficacy of synaptic transmission and the strength of learning and memory. Ciliate cytoskeleton, in the forms of myonemes and axonemes, possibly serve during each engine cycle as conduits for transduced sensory information and as memory-sensitive decoders selectively operating on biochemical messages underlying respective serial contraction and reversal codes prior to behavioral execution. Despite possible differences between higher and lower eukaryotes, learned changes in intracellular Ca^2+^ regulation may thus largely mediate both the classical and quantum computations of ciliates. Learning often improves the information processing efficiency of biological systems. And for ciliates, Ca^2+^-dependent, Hebbian-like tuning of information processing efficiency effects transitions from classical to quantum performance levels resembling the operation of a mechanochemical engine capable of both classical and quantum computational phases during social problem solving (Clark, 2010a,b,c,d,e, 2011a,b,c, 2012b). Taken together, the present findings strongly indicate ciliates and perhaps other types of microbes implement fault-tolerant information processing with mechanosensation and/or additional “sensorimotor” pathways important for expression of learned non-social and social behaviors and capable of signal integration, coincidence detection, and feedback regulation. Phylogenetically ubiquitous calcium response regulatory systems likely play a major role in mediating error-syndrome diagnosis and correction of intracellular information via facilities for digital representation and compliance with heat engine and refrigeration statements derived from thermodynamic laws.

### Conflict of interest statement

The author declares that the research was conducted in the absence of any commercial or financial relationships that could be construed as a potential conflict of interest.

## Appendix

### Shannon information theory for vibration meaningfulness and the content of serial signals during mating games

Calcium-dependent escape behaviors, such as contraction and ciliary reversal, are evoked from ciliates when ciliates perceive a need to avoid or escape mildly noxious mechanical stimuli (Clark, 2010a). This long-time, standard experimental interpretation of ciliate behavior gives the foundation for developing a Shannon information theory for vibration meaningfulness used in the present study and elsewhere. Shannon or classical entropy, $H=-\sum_{n}p_{n}\log_{2}p_{n}$, measures how much information is gained about an event, *E*, of probabilities, *p*_1_ … *p*_*n*_, after that event has been sampled (Shannon, 1948a,b). It also measures the uncertainty about an event *E* before that event has been sampled. Probabilities *p*_*n*_ of event *E* range from zero to one. Because responsiveness, Ω, is the reaction rate (e.g., 0.0–0.1 Hz) or probability of preparedness to react (e.g., 0–1) at a certain rate to an event *E* (Hamilton, 1975; Hamilton et al., 1974; Eisenstein et al., 1982; Eisenstein and Eisenstein, 2006; Clark, 2010a), it serves as the reference to meaningfulness of event *E* for the respondent. This is best understood through the psychophysical Stevens' Law of subjective stimulus intensity: *I* = *K*(*S* − *S*_0_)^*n*^, where *I* is subjective intensity, *K* is a constant, *S* is supratheshold stimulus, *S*_0_ is threshold stimulus, and *n* is an exponent (Stevens, 1958). Stevens' Law measures the perceived or subjective intensity of some stimulus event *E* reported by a respondent. Responsiveness Ω is a respondent's report of its subjective experience with some event *E*. High responsiveness by a respondent indicates the stimulus event is deemed more intense or noxious and low responsiveness by a respondent indicates the stimulus event is deemed less intense or noxious (Eisenstein and Eisenstein, 2006). In mating situations, stimulus intensity corresponds to the aggressiveness of suitors and rivals engaging or attempting to engage in courtship dances, dominance displays, and preconjugal cell-cell touches (Clark, 2010a). Vibrations, previously shown to carry meaningful information content as determined by Stevens' Law of subjective stimulus intensity (Clark, 2010a), simulate the motions and mechanical pressures of conspecifics seeking to conjugate a mating partner and conformed to two-player games beginning in one of two perfect Bayesian equilibria, pooling or separating equilibrium (see Clark, 2010a, c for more details). Honest two-player signaling games often model this kind of ambiguous social setting for animal sexual behavior (Johnston, 1995). One player, the sender (i.e., the ambiguous vibration source), communicates a risky, reliable message of mating prowess not intended to deceive, such as ornate or prudent morphology and behavior. Observing these signals with incomplete knowledge of the sender, the second player, termed the receiver (i.e., the ciliate), appreciates the metabolic and predation costs of the message for the sender, makes a judgment about the sender's fitness quality referenced to those costs, and picks a behavior in reply. The type of sender, its message, and the receiver's reply figure into the payoffs expected and/or procured by both parties (Clark, 2010a, c). Because games start in perfect Bayesian equilibrium, signals played in an honest signaling game must meet four requirements. The first requirement states the receiver must have a probable “belief” about which types of sender communicated a message conditional on the message's content. The second requirement states the action receivers select in reply to any message must maximize their expected utility given their beliefs about the sender. The third requirement states for each type the sender might represent, the sender selects signals that maximizes its utility given the reply chosen by the receiver. The fourth and final requirement states Bayes Rule must hold.

For this study of error diagnosis and correction, as similarly reported elsewhere (Clark, 2010a), event *E* was the occurrence of a block (i.e., series) of three consecutive vibrations and the respondent was a ciliate deciding to avoid or not to avoid the felt vibrations within that block of trials. Vibrations were artificially produced and gave no physical cues to ciliates for them to determine the type of vibration source (e.g., other ciliates of same or different mating type). For every event *E* experienced by a ciliate (i.e., a total of 58 sequential intervals of three consecutive vibrations; see next subsection) that ciliate accordingly reported, Ω, its subjective interpretation of *E*. Ciliates, therefore, provided the subjective context for vibration meaningfulness. When a ciliate increases responsiveness Ω, the vibration series should be perceived by that ciliate to be sufficiently intense to merit avoidance or to play harder-to-get in mating contexts. When a ciliate decreases responsiveness Ω, the vibration series should be perceived by that ciliate to be sufficiently weak to not merit avoidance or to play easier-to-get in mating contexts. Stimulus meaningfulness, *M*, defined as a Shannon information measure, *M* = −∑Ω log_2_ Ω, gave the amount of information expected and acquired by a ciliate about its subjective interpretation of the intensity of some series of three consecutive vibrations. The value of stimulus meaningfulness is mathematically related to Stevens' Law by the equality, Ω = I, and may vary over the range of zero to one. Meaningfulness of a vibration series was uncertain to a ciliate before the series occurred. But level of responsiveness revealed what a ciliate expected about pending vibrations (e.g., less or more harmful or less or more aggressive). The amount of information acquired after experiencing a vibration series revealed what the ciliate learned about the series (e.g., less or more harmful or less or more aggressive). And the Shannon mutual information of stimulus meaningfulness, *M*(*E*_*m*_: *E*_*n*_) = *M(E_m_)* + *M(E_n_)* − *M*(*E_m_*, *E*_*n*_), between separate vibration blocks *E*_*m*_ and *E*_*n*_ with Ω_*m*_ and Ω_*n*_, respectively, revealed how much classical information about the meaningfulness of a previous vibration series was remembered by a ciliate.

The ambiguity of the artificial vibration source to a ciliate lends itself to game analysis of mock social situations taking place in an indistinguishable environment of competing/antagonistic autocrine (same mating type) and paracrine (opposite mating type) mating pheromones (Clark, 2010a). Mock social situations allow an experimental protocol free of confounds introduced by other ciliates (e.g., unresponsive mating types), yet capable of simulating behavioral conditions, such as mechanical pressures, experienced by a single ciliate during preconjugal contacts and courtship and dominance displays (cf. Miyake, 1981). This type of paradigm parallels, in validity and reliability, those used to study animal sexual behavior, where (recorded) real or artificial stimuli, such as sounds and/or images, mimic the presence of conspecifics to evoke courtship and/or dominance displays from test subjects in laboratory or wild environs regardless of pheromone emissions. Accordingly, a two player game of mock social interaction was played by the sender (i.e., the ambiguous vibration source “acting” as or assigned to be the same and opposite mating type of the receiver with equal probability) and the receiver (i.e., each ciliate tested; Clark, 2010a). The sender initiated the game, choosing to transmit a message (i.e., serial vibrations) from a set of probable messages, *M* = {*m*_1_, *m*_2_, …, *m*_*j*_}. The receiver, after perceiving the message, chose to reply from a set of probable actions (i.e., serial contractions or reversals), *A* = {*a*_1_, *a*_2_, …, *a*_*j*_}. For convenience, sets of probable messages and replies formed from only four orthogonal sets of signals representative of net reproductive payoffs (Clark, 2010a). Two types of signals conveyed information about potential individual reproductive benefits, *B*(Ω), and costs, *B*(1 − Ω). And two types of signals conveyed information about potential paired reproductive benefits, (1 − *B*)Ω, and costs, (1 − *B*) (1 − Ω). These signals were simple products of potential reproduction count and responsiveness. All ciliates had equal opportunities for potential solitary asexual binary fission or cooperative sexual-like conjugation and were assumed to be capable of producing two or eight daughter cells of respective probabilities, *B* = 0.2 and 1 − *B* = 0.8. Responsiveness was defined as the probability of a ciliate being prepared, Ω, or not being prepared, 1 − Ω, to make a serial response. Responsiveness for the ambiguous vibration source was always at unity.
